# Changes in the *Sclerotinia sclerotiorum* transcriptome during infection of *Brassica napus*

**DOI:** 10.1186/s12864-017-3642-5

**Published:** 2017-03-29

**Authors:** Shirin Seifbarghi, M. Hossein Borhan, Yangdou Wei, Cathy Coutu, Stephen J. Robinson, Dwayne D. Hegedus

**Affiliations:** 1 Agriculture and Agri-Food Canada, 107 Science Place, Saskatoon, SK S7N 0X2 Canada; 2 Department of Biology, University of Saskatchewan, Saskatoon, Canada; 3 Department of Food and Bioproduct Sciences, University of Saskatchewan, Saskatoon, Canada

**Keywords:** *Sclerotinia sclerotiorum*, *Brassica napus*, Infection, Transcriptome, Necrosis, Effectors, Hydrolytic enzymes, Secondary metabolites, Oxalic acid

## Abstract

**Background:**

*Sclerotinia sclerotiorum* causes stem rot in *Brassica napus*, which leads to lodging and severe yield losses. Although recent studies have explored significant progress in the characterization of individual *S. sclerotiorum* pathogenicity factors, a gap exists in profiling gene expression throughout the course of *S. sclerotiorum* infection on a host plant. In this study, RNA-Seq analysis was performed with focus on the events occurring through the early (1 h) to the middle (48 h) stages of infection.

**Results:**

Transcript analysis revealed the temporal pattern and amplitude of the deployment of genes associated with aspects of pathogenicity or virulence during the course of *S. sclerotiorum* infection on *Brassica napus*. These genes were categorized into eight functional groups: hydrolytic enzymes, secondary metabolites, detoxification, signaling, development, secreted effectors, oxalic acid and reactive oxygen species production. The induction patterns of nearly all of these genes agreed with their predicted functions. Principal component analysis delineated gene expression patterns that signified transitions between pathogenic phases, namely host penetration, ramification and necrotic stages, and provided evidence for the occurrence of a brief biotrophic phase soon after host penetration.

**Conclusions:**

The current observations support the notion that *S. sclerotiorum* deploys an array of factors and complex strategies to facilitate host colonization and mitigate host defenses. This investigation provides a broad overview of the sequential expression of virulence/pathogenicity-associated genes during infection of *B. napus* by *S. sclerotiorum* and provides information for further characterization of genes involved in the *S. sclerotiorum*-host plant interactions.

**Electronic supplementary material:**

The online version of this article (doi:10.1186/s12864-017-3642-5) contains supplementary material, which is available to authorized users.

## Background

*Sclerotinia sclerotiorum* (Lib.) de Bary causes one of the most devastating diseases of canola, stem rot. This pathogen has a wide host-range and can infect more than 400 plant species, including many other important crop plants [1]. This fungus was long considered to be a prototypical necrotrophic pathogen whereby immediately upon host cuticle penetration a highly aggressive pathogenic phase ensues where acids and hydrolytic enzymes are liberated in advance of the invading mycelia with a trailing saprophytic phase that supports sclerotia formation [2]. Recent studies, however, have provided evidence for a brief biotrophic phase occurring within the apoplastic space immediately after cuticle penetration and the pathogen may, therefore, be more accurately classified as a hemi-biotroph [3]. Transition between these various developmental and pathogenic phases is governed by physical and metabolic cues including detection of contact with hard surfaces [4], glucose levels [5], cAMP levels [6], pH [7] and oxidative stress [8]. Communication between the associated signaling pathways is critical and involves numerous protein kinases [9–11] and phosphatases [12, 13].

Much of the research on the molecular mechanisms of virulence in *S. sclerotiorum* has focused on oxalic acid (OA) which plays various roles during several stages of the infection [14]. OA suppresses the oxidative burst and callose deposition during the early stages of the infection [15]. Suppression of host defenses by OA during the biotrophic phase is thought to allow sufficient time for the pathogen to establish itself in the host as a prelude to mycelial ramification [3]. Subsequently, OA induces the production of host reactive oxygen species (ROS), which in turn leads to host cell death [16]. As a central player in *S. sclerotiorum* pathogenesis, it is not surprising that plants expressing oxalate-degrading enzymes exhibit increased resistance to this pathogen [17]. Pathogen-derived ROS generated through NADPH oxidase activity are associated with appressoria formation and sclerotial development, as well as oxalic acid synthesis [8]. Catalase (*SCat1*) [18] and superoxide dismutase (*SsSodI*) [19] appear to modulate the deleterious effects of these compounds internally. The apoptosis inhibitor, BAX inhibitor-1 (*SsBI1*), is also required for full virulence and was postulated to prevent hyphal apoptosis resulting from exposure to host-derived ROS [20].

The production of numerous types of hydrolytic and cell wall degrading enzymes (CWDE) facilitates host cuticle penetration, lesion expansion and tissue maceration [4, 21, 22]. Although pectinolytic CWDEs, such as polygalacturonases (SsPG1, SsPG3, SsPG5 and SsPG6), have captured more attention as the main group of hydrolytic enzymes involved in *S. sclerotiorum* virulence [23], non-pectinolytic enzymes like proteases, cellulases and glucoamylases also contribute to the infection process in this fungus [21].

Several other factors are known to contribute to *S. sclerotiorum* pathogenicity and host interactions. Both γ-glutamyl transpeptidase (*SsGgt1*) and compound appressorium formation-related protein 1 (Ss*-*Caf1) influence the production of compound appressoria and subsequent host penetration, but also development of sclerotia [24, 25]. A secreted integrin-like protein (SSITL) inhibits the deployment of plant defenses through the jasmonic/ethylene signaling pathways [26] and a chorismate mutase (*SsCm1*) may function similarly to suppress plant defense responses during the biotrophic phase [27]. Host chemical defenses may be inactivated by inducible detoxification systems [28], while other proteins, such as SsPemG1 (protein elicitor from *Magnaporthe grisea*), are recognized by the host and induce defenses [29]. *SsNEP1* and *SsNEP2* encode necrosis and ethylene-inducing like proteins (NLP), which induce necrosis in host tissues [30], as does cutinase [31]. A gene (SS1G_00263, ssv263) encoding a hypothetical protein with unknown mode of action is a virulence factor in *S. sclerotiorum* [32].

Transcriptomics and proteomics approaches have been used to gain insight into molecular interaction of *S. sclerotiorum* with its various hosts. Expressed sequence tag (EST) analysis was used to identify genes associated with pathogenesis by comparing the transcriptome of *S. sclerotiorum* grown on artificial medium to that during infection of *Brassica napus* [33]. A similar approach was used to identify genes expressed during different stages of *S. sclerotiorum* development on this host [34], which was later supported by proteomics analysis [35]. Subsequently, microarray [36] and RNA-Seq analysis [37] was used to explore the *B. napus* responses to *S. sclerotiorum*. The release of the *S. sclerotiorum* genome sequence [38] in combination with next generation sequencing has allowed for in-depth analysis of the *S. sclerotiorum* - pea [39], *S. sclerotiorum* - *Phaseolus vulgaris* [40] and *S. homoeocarpa* - creeping bentgrass [41] pathosystems. Proteomic analysis of exudates from liquid cultures has identified several secreted proteins that may be involved in aspects of pathogenesis [42]. Bioinformatic studies revealed that *S. sclerotiorum* has the potential to secrete a large number of proteins, many of which have the potential to influence host-pathogen interactions [43, 44].

While significant progress has been made in the characterization of individual *S. sclerotiorum* virulence and pathogenicity factors, a gap exists in our understanding of how the transcriptome is deployed throughout the course of *S. sclerotiorum* infection on a host plant. In this study, we used RNA-Seq analysis to comprehensively catalogue genes that were expressed and up-regulated during infection of *B. napus*, with a particular focus on the events occurring early in the infection. This work provided new insight into *S. sclerotiorum* pathogenesis through examination of the sequential expression of virulence and pathogenicity genes during infection establishment.

## Methods

### Biological materials and disease assay

*S. sclerotiorum* isolate 1980 (Ss1980) was used in this study as the genome sequence of this strain is available [38]. The doubled haploid *B. napus* cultivar DH12075 for which a genome sequence is available (Parkin, unpublished) was used as the host plant. Ss1980 was grown on minimal salts-glucose (MS–Glu: 2 g/L NH_4_NO_3_, 1 g/L KH_2_PO_4_, 0.1 g/L MgSO_4_·7H2O, 0.5 g/L yeast extract, 3 g/L DL-malic acid, 1 g/L NaOH, supplemented with 1% glucose) medium and mycelia were used for inoculation as described earlier [23]. One gram of mycelia (wet weight) was spread over a 5-cm diameter circle on a detached leaf of a four week old plant and incubated in a sealed and humidified tray at room temperature. The experiment was conducted with three biological replicates. Samples collected from the fungal isolate grown in culture and on plants at 1, 3, 6, 12, 24 and 48 h post-inoculation (hpi) were subjected to RNA-Seq analysis.

### RNA extraction, library preparation and Illumina sequencing

Fungal mats and the infected plant tissues beneath it were flash-frozen in liquid nitrogen and stored at -80 °C. The samples were ground to a fine powder with an RNAse-free mortar and pestle precooled with liquid nitrogen. Total RNA was extracted using an Illustra RNAspin mini RNA isolation kit (Illumina, San Diego, USA). RNA quantity and quality was assessed using a Qubit fluorometry assay (Invitrogen Corp., Carlsbad, CA, USA) and an Agilent 2100 Bioanalyzer (Agilent Technologies, Palo Alto, CA, USA), respectively. Libraries were prepared using a Truseq stranded mRNA kit (Illumina, San Diego, USA) following the manufacturer’s instructions. Sequencing was conducted on an Illumina MiSeq sequencing system using the Illumina MiSeq reagent kit V3 (Illumina, San Diego, USA) following the manufacturer’s instructions.

### Data analysis

*S. sclerotiorum* transcripts available in the database (http://www.broadinstitute.org/annotation/genome/sclerotinia_sclerotiorum/MultiHome.html) were used as a reference for mapping the short reads using CLC Genomics Workbench 7.0.4 (http://www.clcbio.com). Gene expression was estimated by extracting read counts as integers from the CLC Genomics alignments. The count data were normalized to generate effective library sizes using the scaling method Trimmed Means of Means values (TMM) [45]. Statistical analysis was performed with these data using a generalized linear model linked to the negative binomial distribution performed using the EdgeR package [45]. Pair-wise analyses were performed to assess differential gene expression using the control library as a common reference standard. Genes were considered differentially expressed where the probability after adjustment for multiple hypothesis testing [false discovery rate (FDR)] was less than 0.05. The extent of the observed differential expression was considered meaningful if the fold change exceeded a factor of two. Finally, all significantly up-regulated genes at different sampling times were assigned a functional classification using the BLAST2GO plugin (v1.4.4) in the CLC Genomics Workbench 8.0.1 for functional annotation using Interpro and the NCBI refseq protein database. Gene ontology (GO) terms for each gene were extracted. The results were filtered to remove top-level annotations and apply the GO-slim categorization from *Aspergillus* in order to summarize the results. Blast2GO ran ANNEX [46] to add implicit GO terms for a more complete annotation. Finally, Blast2GO was used to calculate the abundance of GO classifications for the significantly up-regulated genes for each time point. Candidate genes were categorized into different groups based on known functions of orthologous genes in other fungi.

### Validation of RNA-Seq analysis using droplet digital PCR (ddPCR)

cDNA was synthesized from 1μg of total RNA using the iScript Reverse Transcription Supermix for RT-qPCR kit (Bio-Rad, CA, USA) following the manufacturer’s instructions. The ddPCR was conducted with three biological replicates using a droplet digital PCR QX200 system (Bio-Rad, CA, USA). No-reverse transcriptase (no-RT) controls were also used to detect genomic DNA contamination. Primers and probe for each gene were designed using PrimerQuest tool (IDT) and all probes were labeled with fluorescein amidite (FAM), except for the reference gene (β-tubulin) which was labeled with hexachloro-fluorescein (HEX). Sequences and details of primers and probes have been provided in Additional file 1: Table S1. The ddPCR reaction mixtures (20 μl) contained 1X ddPCR supermix (Bio-Rad Laboratories, Hercules, CA), 900 nM of each primer, 250 nM of probe and 4 μl of 1:100 diluted cDNA. The PCR was performed in a C1000 Touch Thermal Cycler (Bio-Rad, CA, USA) with the following cycling conditions: 95°C for 10 min; 50 cycles of 94°C for 30 s, 53°C for 75 s, Ramp 2°C/s; 98°C for 10 min. The droplet generation and reading for ddPCR were conducted using a Droplet Generator and Reader (Bio-Rad QX200 system), respectively, according to the manufacturer’s instructions. The gene expression ratio was calculated by QuantaSoft droplet reader software (Bio-Rad). The expression of the β-tubulin gene (SS1G_ 04652) is constant during the infection, confirming its validity as a reference gene. The fold change in the expression of each gene was calculated by dividing the ratio of the target to the reference (β-tubulin) gene for each time point by the ratio from the sample collected from fungi grown in culture (i.e. time zero).

## Results and discussion

### RNA sequencing

Illumina sequencing (RNA-Seq) was used to conduct sequential transcriptional profiling in order to identify differentially expressed genes involved in *S. sclerotiorum* establishment on and subsequent infection of *B. napus*. Mycelia were collected from liquid media immediately prior to inoculation (time 0) and at 1, 3, 6, 12, 24 and 48 hpi. The number of reads per each biological replicate per each time point is shown in Additional file 2: Table S2. A total of 40,210,134 paired-end reads were generated. Reads mapped to 14,503 of the 14,522 predicted genes (99% of total reference transcripts), indicating sufficient sequencing depth. Genes with expression ratios greater than two relative to the inoculum grown on MS-Glu medium and a false discovery rate (FDR) *p*-value correction of < 0.05 were considered to be up-regulated. The numbers of up-regulated genes were 584, 582, 526, 371, 822 and 1283 at 1, 3, 6, 12, 24 and 48 hpi, respectively, ranging from 2.6% to 8.8% of total expressed genes. The RNA-Seq data was submitted to NCBI (accession # GSE83935) and the list of up-regulated genes with BLAST2GO annotation can be found in Additional file 3: Table S3.

**Fig. 1:**
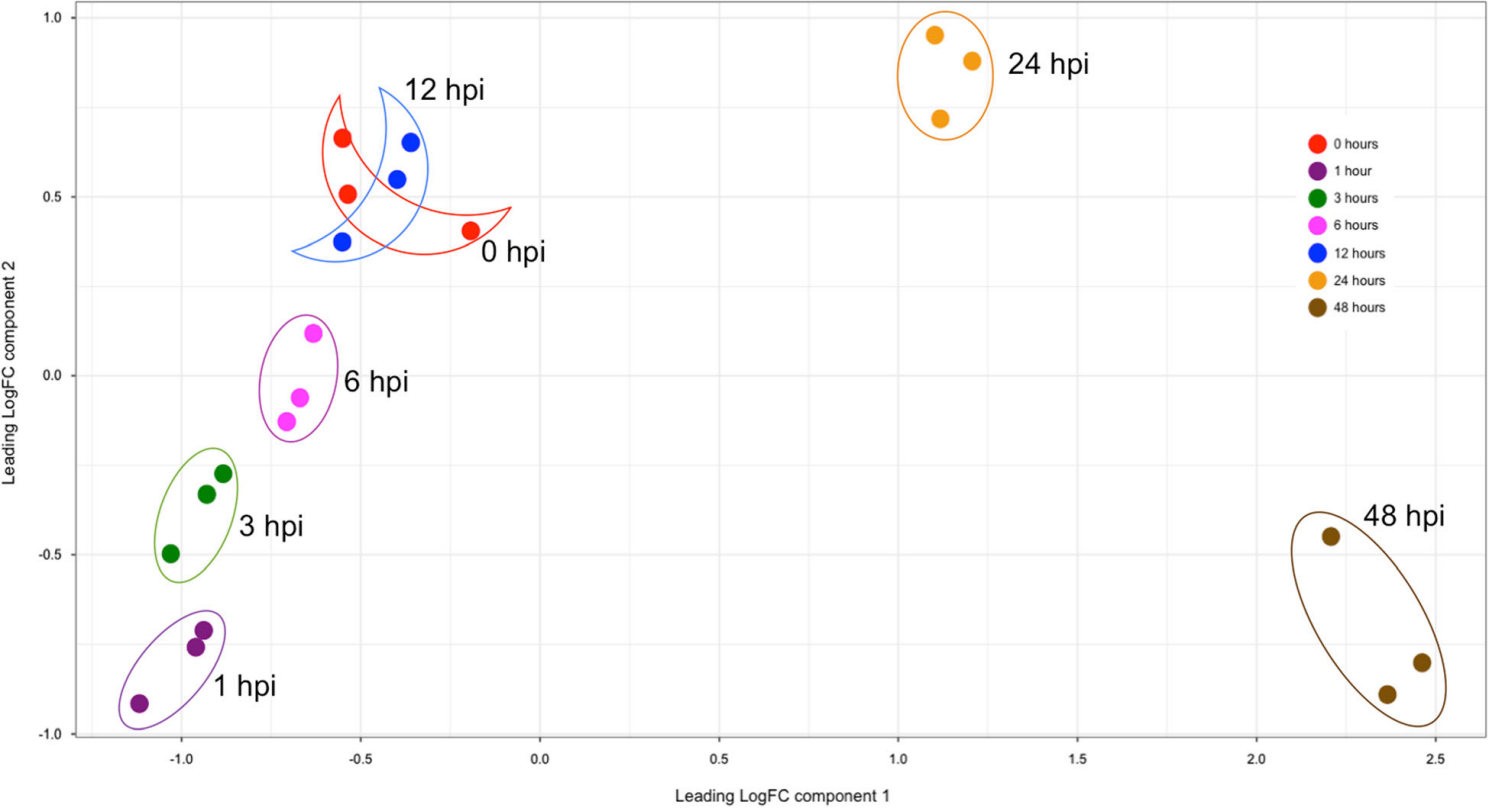
Principal component analysis showing the relatedness among the gene expression patterns of samples used for RNA-Seq analysis. Samples were collected from the inoculum (0 time) and at 1, 3, 6, 12, 24 and 48 h post *Sclerotinia sclerotiorum* inoculation on *Brassica napus* leaves in three biological replicates

To confirm the relatedness of the three biological replicates and the accuracy of the RNA-Seq analysis, principal component analysis (PCA) was conducted (Fig. 1). Individual replicates of each time point clustered together, indicating a high degree of similarity in the expression profiles and low biological variability among the experimental replicates. Of the early infection time points sampled (1-12 hpi), the 1 hpi sample was most different from the zero time point with successive early time points becoming increasingly more similar to the inoculum. PCA also showed a clear distinction between the *S. sclerotiorum* transcriptomes at 24 and 48 hpi compared to the other time points which was due to a significant increase in both the number and types of genes expressed at these time points.

### Gene ontology analysis of up-regulated genes

**Fig. 2:**
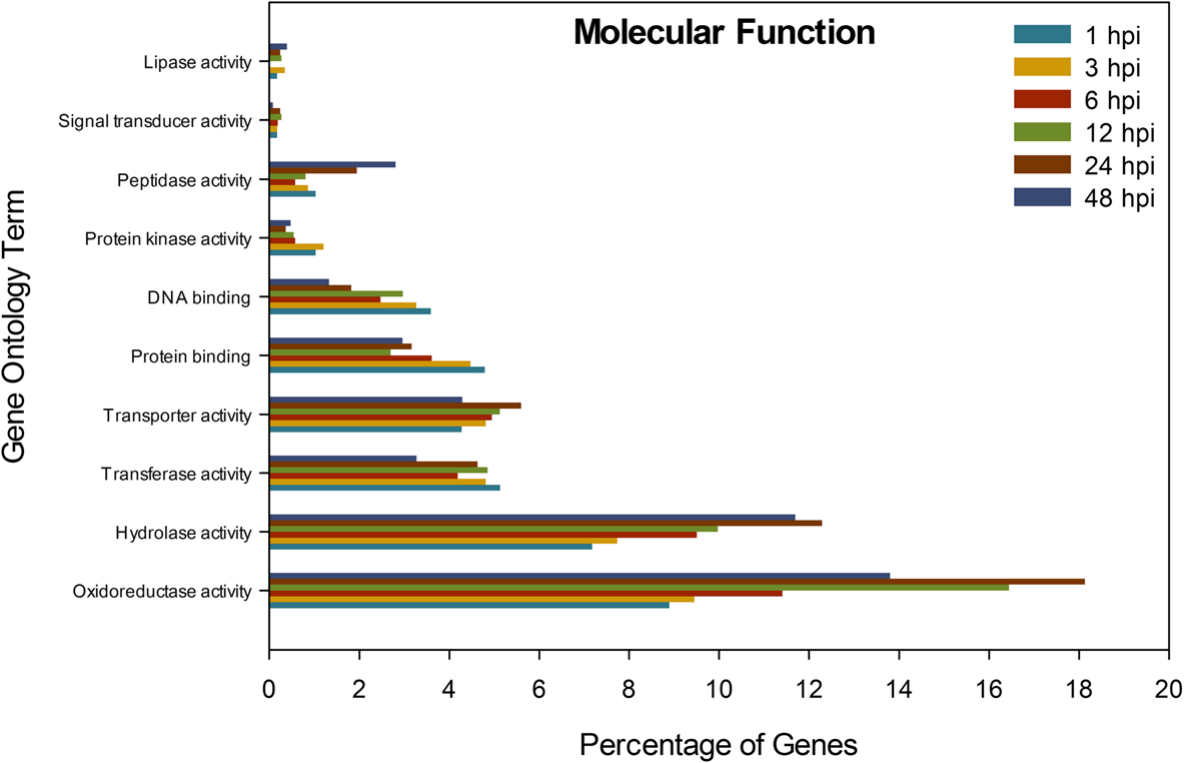
Percentage of genes encoding enzymes assigned to Molecular Function subcategories (indicated) that were induced upon *Sclerotinia sclerotiorum* infection of *Brassica napus*. Gene ontology analysis was conducted using Blast2Go software. hpi, hours post-inoculation

**Fig. 3:**
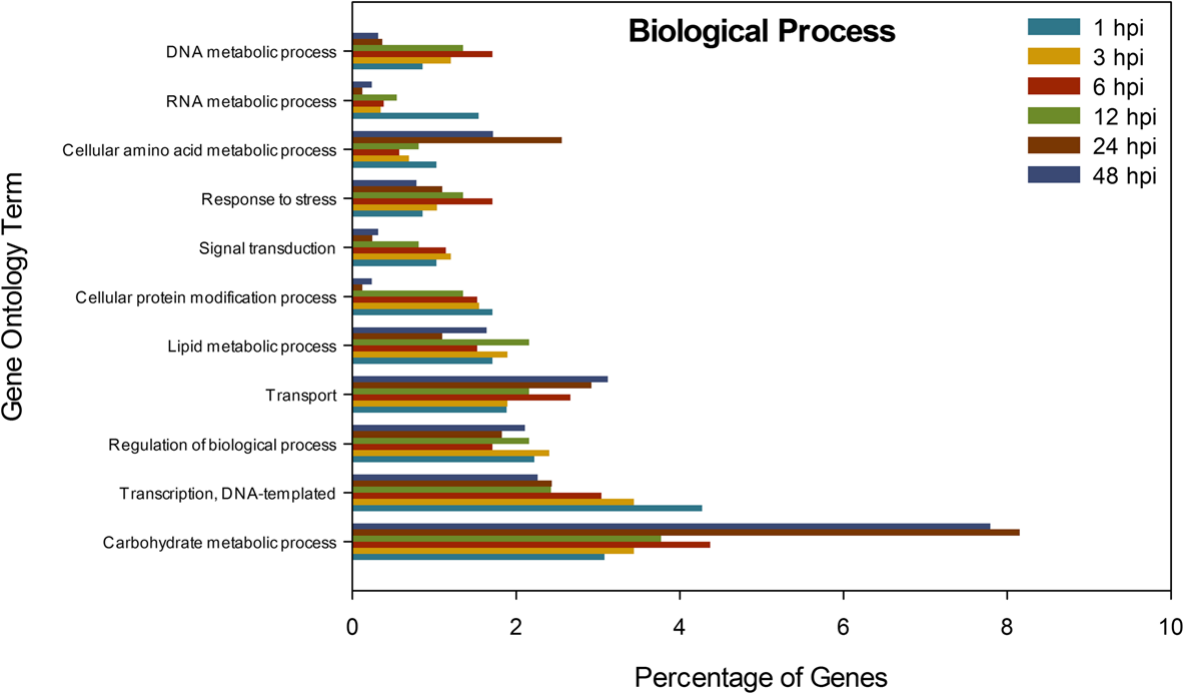
Percentage of genes encoding enzymes assigned to Biological Process subcategories (indicated) that were induced upon *Sclerotinia sclerotiorum* infection of *Brassica napus*. Gene ontology analysis was conducted using Blast2Go software. hpi, hours post-inoculation

To obtain an overall view of the genes involved in *S. sclerotiorum* infection, gene ontology (GO) analysis of the up-regulated genes was performed. Blast2GO using different forms of annotations, including Interpro, GO-slim, enzyme code and Annex, was used to calculate the abundance of GO classifications in each of these ontology categories, molecular function (Fig. 2) and biological processes (Fig. 3) for each time point. In total, 25%, 25%, 26%, 22%, 15% and 18% of the up-regulated genes at the 1, 3, 6, 12, 24 and 48 hpi sampling times, respectively, were annotated as encoding proteins with unknown functions and therefore could not be assigned to a GO category. The majority of up-regulated genes in the molecular function group fell into the subcategories of oxidoreductase and hydrolase activity in all sampling times. The highest proportion of up-regulated genes belonging to oxidoreductase and hydrolase activity subcategories was at 24 hpi, and declined at 48 hpi coincident with the appearance of visible necrotic lesions. For the other molecular function subcategories, genes classified as encoding proteins with transferase, transporter, protein binding, DNA binding, protein kinase and signal transducer activity, the lowest proportion of up-regulated genes was at 48 hpi, suggesting a decrease in the expression of these genes after the start of necrotic stage.

The majority of up-regulated genes within the biological processes category belonged to carbohydrate metabolic process subcategory. The highest proportion of genes within this subcategory was found at 24 hpi, with a notable increase compared with 12 hpi, and a decline by 48 hpi, similar to the hydrolytic activity subcategory. This supports the connection between carbohydrate metabolic activity and hydrolytic enzyme activity as these processes work in concert to supply nutrients and energy for mycelial proliferation and to facilitate the transition to the necrotrophic phase occurring beyond 24 hpi in this experimental system.

The highest proportion of up-regulated genes related to transcription and signal transduction was observed at 1 and 3 hpi, respectively. This is expected as the pathogen needs to modulate the expression of a wide variety of developmental and metabolic genes during the early stages of the infection as it penetrates the host cuticle and then establishes within the host. Beyond this, the 6 hpi time point had the highest percentage of up-regulated genes involved in response to stress. This is likely a response to the exposure of the pathogen to host plant defense mechanisms. Kabbage et al. [3] proposed that a brief biotrophic phase occurs soon after cuticle penetration where the pathogen either remains undetected or compromises/tolerates host defenses. The induction of stress-related genes soon after cuticle penetration would alleviate some of the effects of these stress conditions and allow host colonization. Interestingly, after events leading to cuticle penetration (1 hpi), dramatic changes in gene expression appear to subside as the overall gene expression profiles at 3, 6 and 12 hpi become increasingly similar to that of the inoculum at time 0 (Fig. 1). This period may constitute the biotrophic phase which is followed by a mycelial ramification phase (24 hpi) and finally a necrotic phase (48 hpi), each of which have unique expression profiles. This is in accordance with the gene expression profiles of *B. cinerea* on *Arabidopsis thaliana* leaves where three distinct groups of genes were identified, these being early, outset of colonization and complete colonization, based on expression patterns [47].

### Validation of RNA-Seq analysis using droplet digital PCR (ddPCR)

**Fig. 4:**
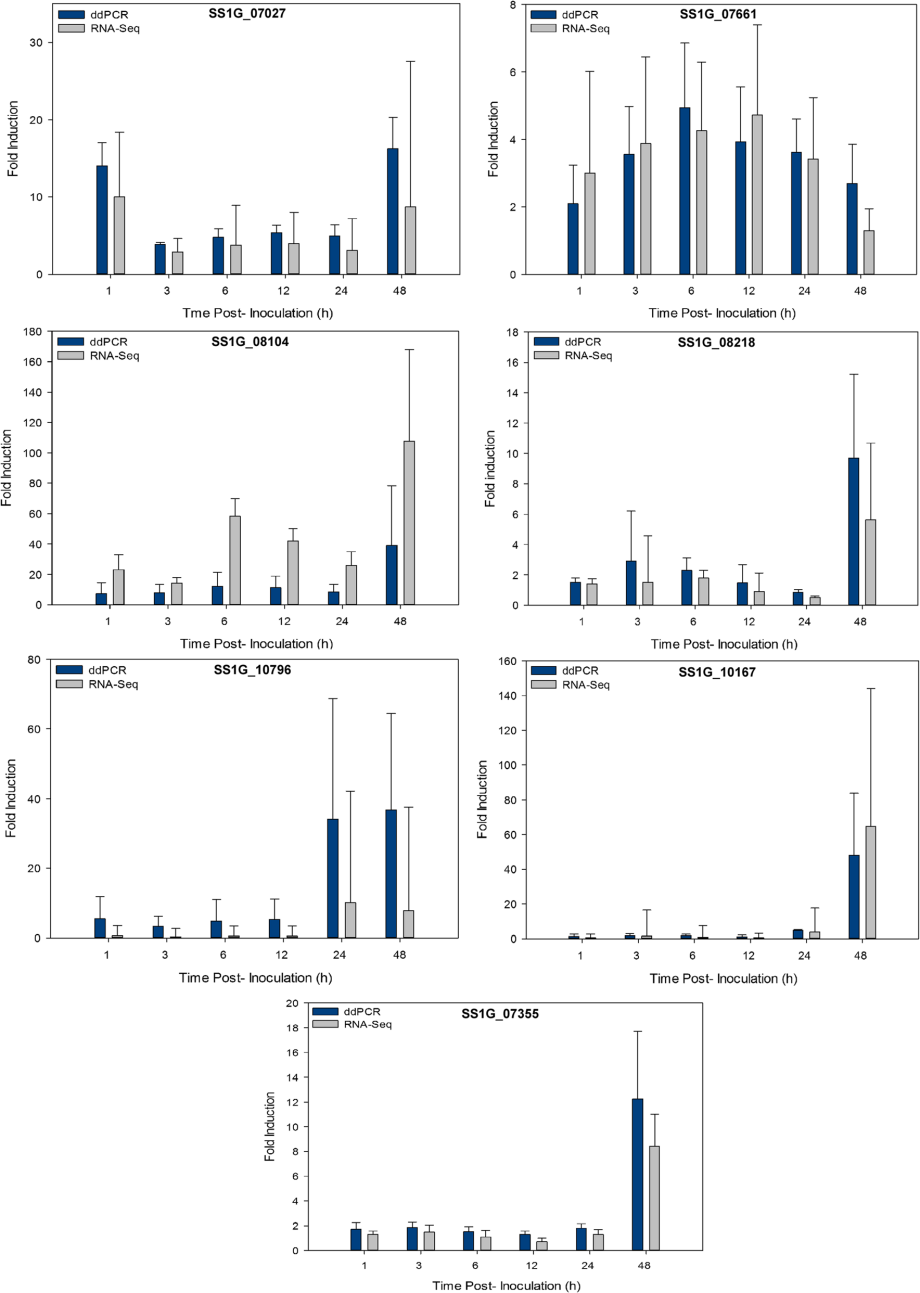
Expression of various *Sclerotinia sclerotiorum* genes during infection of *Brassica napus* as determined by RNA-Seq and droplet digital PCR (ddPCR) analysis. Histograms show the relative expression level (fold change) and are reported as means and standard errors of three biological replicates for hour post-inoculation (hpi)

Three different types of genes from the RNA-Seq data list were selected for validation, including three highly expressed genes that were induced in most of the sampling times (SS1G_07027, SS1G_07661 and SS1G_08104 genes encoding a hypothetical protein, cutinase and acetylxylan esterase, respectively), three that were not induced during the sampling time points (SS1G_14133, SS1G_02486 and SS1G_05839 genes encoding SSITL, SsCaf1 and SsBi1, respectively) and four well-characterized *S. sclerotiorum* genes (SS1G_08218, SS1G_10796, SS1G_10167 and SS1G_07355 encoding oxaloacetate acetyl hydrolase (OAH), oxalate decarboxylase, SsPG1 and the Pac1 transcription factor, respectively). The ddPCR analysis generated patterns of expression for the induced genes that were very similar to that predicted from the RNA-Seq data (Fig. 4). Only one out of the seven genes (SS1G_08104) tested showed a slightly different trend between ddPCR and RNA-Seq. Previous work showed about 90% correlation between qPCR and RNA-Seq [48] suggesting that slight variation between the two methods is expected, but is generally negligible. Furthermore, for the three genes that were not induced in the RNA-Seq analysis, the fold-change in expression did not exceed a factor of 2 when examined by ddPCR, providing additional evidence that they were not induced in the current study (data not shown).

### Functional classification of the genes

To simplify the exploration of genes expressed during the course of *S. sclerotiorum* infection, genes were clustered based on expression patterns (Additional file 4: Figure S1) and categorized based on their functional annotation and predicted roles in pathogenicity or virulence; these are discussed below.

#### 1. Hydrolytic enzymes

The current study revealed that a large number of the genes induced during infection encoded enzymes with hydrolytic activity (Table 1). The largest group of these genes encoded carbohydrate-active enzymes (CAZymes). Most of the CAZyme genes predicted in the *S. sclerotiorum* genome by Amselem et al. [38] were up-regulated during infection in this study and in the study by Lyu et al., [49] which examined different *S. sclerotiorum* developmental stages. These results consistently support the important role of these enzymes during infection. The majority of the genes encoding CAZymes reported in the current study were from the glycoside hydrolase (GH) and carbohydrate esterase (CE) families. The expression of numerous genes encoding GH and CE enzymes in the current study and in a similar study of *Sclerotinia homoeocarpa* reflects the ability of these pathogens to infect a wide range of plant hosts [41]. These are discussed in more detail below.

**Table 1:** Description and expression of up-regulated genes encoding hydrolytic enzymes

Gene ID	Description^a^	Expression level (hpi)^b^					
		1	3	6	12	24	48
*1. Lipid Degradation*							
SS1G_07661	cutinase (SsCuta)	3	3.9	4.3	4.7	3.4	-
SS1G_13386	cutinase	-	-	-	-	14.9	29.6
SS1G_12907	cutinase	-	-	-	-	29.7	236
SS1G_09557	lipase/esterase	3.4	5.5	6.5	6.1	2.6	-
SS1G_11473	lipase/esterase	2.2	-	-	-	-	3.2
SS1G_05990	lipase/esterase	-	-	-	-	2.2	-
SS1G_00767	lipase/esterase	-	-	-	-	-	34.4
SS1G_03597	lipase/esterase	-	-	-	-	-	5.1
SS1G_01849	lipase/esterase	-	-	-	-	-	2.7
SS1G_08869	lipase class 3	-	-	-	-	-	2.8
SS1G_14146	extracellular lipase	-	-	-	-	31.5	38.7
SS1G_04490	extracellular lipase	-	-	-	-	2.5	3
SS1G_00877	extracellular lipase	-	-	-	-	-	4.3
SS1G_14441	triacylglycerol lipase	-	-	-	-	5	6.9
SS1G_13982	triacylglycerol lipase	-	-	-	-	4.8	27.5
SS1G_01472	triacylglycerol lipase	-	-	-	-	3.6	13.1
SS1G_03007	alpha beta-hydrolase (esterase)	-	2.6	2.6	-	-	-
SS1G_09718	alpha beta-hydrolase (esterase/lipase)	-	2.1	2.9	-	-	-
SS1G_01703	alpha beta-hydrolase (esterase/lipase)	-	3.9	3.5	2.2	3.8	6.5
SS1G_13361	alpha beta-hydrolase (esterase/lipase)	-	-	-	2.3	3.2	2.2
SS1G_11402	alpha beta hydrolase (esterase/lipase)	-	-	-	3.6	4.8	3.7
SS1G_13263	alpha beta hydrolase (esterase/lipase)	-	-	-	-	3.8	3.8
SS1G_08133	alpha beta-hydrolase (esterase/lipase)	-	-	-	-	-	2.5
SS1G_02163	alpha beta-hydrolase (esterase/lipase)	-	-	-	-	-	12.5
SS1G_01953	GDSL lipase acylhydrolase family protein	3.6	4	5	4.9	4.8	5.6
SS1G_13560	GDSL lipase acylhydrolase family protein	-	-	-	-	2.7	2.7
SS1G_06389	GDSL lipase acylhydrolase family protein	-	-	-	-	-	2.7
SS1G_02708	cellulose-binding GDSL lipase	-	-	-	-	3.3	7.8
SS1G_14289	cellulose-binding GDSL lipase	-	-	-	-	5.3	6.6
SS1G_03610	cellulose-binding GDSL lipase	-	-	-	-	5.1	22.4
SS1G_04592	cellulose-binding GDSL lipase	-	-	-	-	11.8	9.4
SS1G_11930	carboxylesterase/lipase (cholinesterase)	4	3.9	8.9	5.2	-	-
SS1G_00376	carboxylesterase/lipase (cholinesterase)	-	-	-	-	4.4	9.2
SS1G_09613	carboxylesterase/lipase (cholinesterase)	-	-	-	-	-	4.7
SS1G_04422	carboxylesterase/lipase (cholinesterase)	-	-	-	-	-	3.2
SS1G_11853	carboxylesterase/lipase (cholinesterase)	-	-	-	-	-	3
SS1G_04030	lysophospholipase	-	-	3.6	-	2.3	-
SS1G_10482	lysophospholipase	-	-	-	-	-	4.1
*2. Polysaccharide Degradation*							
*2.1 Cellulose*							
SS1G_08493	beta-1,4-endo-glucanase	2.6	-	-	-	-	-
SS1G_00891	beta-1,4-endo-glucanase (cellulase)	-	4.5	5.8	3.5	-	3.8
SS1G_01485	beta-glucanase	-	-	-	-	-	2.1
SS1G_09365	endo-glucanase	-	-	-	-	2.7	-
SS1G_03387	endo-glucanase	-	-	-	-	5.3	41.4
SS1G_08837	endo-glucanase	-	-	-	-	10.3	36.4
SS1G_04945	endo-glucanase	-	-	-	-	-	2.3
SS1G_01828	endo-glucanase	-	-	-	-	-	2.6
SS1G_09821	endo-glucanase	-	-	-	-	-	144
SS1G_03041	endo-glucanase	-	-	-	-	-	292
SS1G_00321	endo-glucanase	-	-	-	-	-	4.2
SS1G_00471	endo-glucanase	-	-	-	-	-	2.1
SS1G_06037	exo-glucanase	-	3	3.2	-	9.9	12.3
SS1G_02334	exo-glucanase (cellobiohydrolyase)	-	-	-	-	3.8	-
SS1G_09020	exo-glucanase	-	-	-	-	15.1	79
SS1G_00892	exo-glucanase	-	-	-	-	5.4	22.2
SS1G_09118	exo-glucanase	-	-	-	-	-	2.5
SS1G_02245	exo-glucanase	-	-	-	-	-	3.3
SS1G_13872	exo-glucanase	-	-	-	-	-	7.6
SS1G_02501	Concanavalin A-like lectin/glucanase	5.1	8.7	6.1	3.1	-	-
SS1G_08907	glycoside hydrolase (beta-glucanase)	2.6	-	-	-	-	-
SS1G_07863	cellobiose dehydrogenase	3.7	-	-	-	-	22.1
SS1G_05151	cellobiose dehydrogenase	-	-	-	-	5.9	6.6
SS1G_05118	beta-glucosidase	2.4	-	-	-	-	-
SS1G_01662	beta-glucosidase	-	-	-	-	2.8	9.5
SS1G_09366	beta-glucosidase	-	-	-	-	2.8	8
SS1G_06304	beta-glucosidase	-	-	-	-	2.6	6.8
SS1G_07847	beta-glucosidase	-	-	-	-	3.1	4.7
SS1G_07146	beta-glucosidase	-	-	-	-	7.4	18.7
SS1G_13255	beta-glucosidase	-	-	-	-	40.4	54.8
SS1G_05368	beta-glucosidase	-	-	-	-	3.2	4.6
SS1G_12622	beta-glucosidase	-	-	-	-	-	2.1
SS1G_07162	beta-1,4-glucosidase	-	-	-	-	-	2.3
SS1G_09129	beta-glucosidase	-	-	-	-	-	13.1
SS1G_04264	beta-glucosidase	-	-	-	-	-	2.2
SS1G_01021	beta-glucosidase	-	-	-	-	-	5.2
*2.2 Pectin*							
SS1G_10167	endo-polygalacturonase (SSPG1)	-	-	-	-	3.7	64.6
SS1G_10698	endo-polygalacturonase (SSPG3)	-	-	-	-	-	5.5
SS1G_05832	exo-polygalacturonase	4.1	6.5	-	-	4.2	32.4
SS1G_02553	exo-polygalacturonase (exoPG2)	-	-	-	-	-	22.6
SS1G_04207	exo-polygalacturonase (exoPG1)	-	-	-	-	14.6	168
SS1G_12057	exo-polygalacturonase	-	-	-	-	-	4.2
SS1G_03540	pectin lyase	-	-	-	-	4.5	3.3
SS1G_10071	pectin lyase	-	-	-	-	-	6.2
SS1G_14449	pectin lyase	-	-	-	-	-	2.4
SS1G_04551	pectin methylesterase	-	-	-	-	11.6	35.8
SS1G_00332	pectin methylesterase	-	-	-	-	33.9	178
SS1G_03286	pectin methylesterase	-	-	-	-	4.8	16.5
SS1G_00468	pectin methylesterase	-	-	-	-	-	21.4
SS1G_04095	rhamnogalacturanan acetylhydrolase	2.8	6.2	7.7	5.3	2.7	-
SS1G_12048	rhamnogalacturanan acetylhydrolase	-	-	-	-	-	33.5
SS1G_11992	rhamnogalacturonan acetylesterase	-	-	-	-	-	37.5
SS1G_09857	rhamnogalacturonyl hydrolase	-	-	-	-	-	8.6
SS1G_12964	alpha-l-rhamnosidase	-	-	17.5	-	-	-
SS1G_13501	alpha-l-rhamnosidase	-	-	-	-	3.1	21.2
SS1G_04541	alpha-l-rhamnosidase	-	-	-	-	2	8.3
SS1G_08229	rhamnogalacturonase	2.1	4.5	4.5	2.6	-	-
SS1G_07039	rhamnogalacturonase	-	-	-	-	-	6
SS1G_04552	endo-xylogalacturonan hydrolase	-	-	-	-	7.1	15.9
*2.3 Hemicellulose*							
SS1G_12191	endo-1,4-beta-xylanase	-	5.3	7.9	4.6	6.4	25
SS1G_10092	endo-beta-xylanase	-	-	-	-	-	155
SS1G_03618	endo-beta-xylanase	-	-	-	-	-	451
SS1G_07749	endo-beta-xylanase	-	-	-	-	-	56.7
SS1G_05140	xylanase	3.6	3.5	4.2	4.3	3.4	8.1
SS1G_08104	acetylxylan esterase	23	14.2	58.3	42.1	26	107
SS1G_05434	acetylxylan esterase	-	-	-	-	4	11.8
SS1G_00746	beta-mannosidase	-	-	-	-	6.6	42.3
SS1G_05977	beta-mannosidase	-	-	-	-	3.6	6.4
SS1G_08208	endo-1,4-beta-mannosidase	-	-	-	-	3.6	8.9
SS1G_08118	alpha-xylosidase	-	-	-	-	4.5	3.8
SS1G_09367	alpha-xylosidase	-	-	-	-	2.2	-
SS1G_11535	alpha-fucosidase	-	-	-	-	-	2.5
SS1G_04662	alpha-galactosidase	-	-	-	-	4.5	3.9
SS1G_03386	alpha-galactosidase	-	-	-	-	-	3.7
SS1G_07904	feruloyl esterase	-	-	-	-	3.6	12
SS1G_02462	alpha-l-arabinofuranosidase	-	-	-	-	5.3	21
SS1G_03602	alpha-l-arabinofuranosidase	-	-	-	-	6.3	17.6
*2.4 Arabinogalactans*							
SS1G_01216	arabinogalactan endo-beta-galactosidase	4.1	9.6	11.9	7.2	4.1	-
SS1G_11585	arabinogalactan endo-beta-galactosidase	-	-	-	-	-	19.3
SS1G_02618	galactan 1,3-beta-galactosidase	-	-	-	-	5.7	11
SS1G_10842	beta-galactosidase	4.1	2.5	2.4	-	2.2	5.8
SS1G_11763	beta-galactosidase	-	-	-	-	4.7	-
SS1G_01572	beta-galactosidase	-	-	-	-	6.8	18.3
SS1G_03647	beta-galactosidase	-	-	-	-	3.5	6.9
SS1G_02781	beta-galactosidase	-	-	-	-	-	2.7
SS1G_09866	1,6-beta-galactanase	-	4.8	4.5	3.1	3	12.4
SS1G_11922	arabinan endo-1,5-alpha-L-arabinosidase	-	-	-	-	144.3	245
SS1G_01238	beta-D-glucuronidase	7.6	-	4.7	-	-	-
SS1G_02620	beta-glucuronidase	-	-	-	-	3.5	8.2
*2.5 Lignin*							
SS1G_04196	dihydrogeodin oxidase/laccase	-	3.1	2.6	4.6	-	-
SS1G_06365	dihydrogeodin oxidase/laccase	-	3.1	5.7	4.1	-	3.4
SS1G_05112	dihydrogeodin oxidase/laccase	-	-	-	-	-	6
*2.6 Starch*							
SS1G_01776	alpha-amylase	-	-	2.1	-	-	-
SS1G_11100	alpha-amylase	-	-	-	-	6.6	5.2
SS1G_01083	alpha-glucosidase	2.4	4.7	3	-	4.6	5.3
SS1G_01005	alpha-glucosidase	-	-	-	-	9.2	7.2
*2.7 Mannans*							
SS1G_10867	endo-1,6-alpha-mannosidase	-	-	-	-	6.4	-
SS1G_04468	endo-1,6-alpha-mannosidase	-	-	-	-	7.5	25.3
SS1G_12937	endo-1,6-alpha-mannosidase	-	-	-	-	-	10.4
SS1G_05110	endo-1,6-alpha-mannosidase	-	-	-	-	-	3.5
SS1G_11579	endo-1,6-alpha-mannosidase	-	-	-	-	-	3.5
SS1G_09229	alpha 1,2 mannosidase	-	-	-	-	55	40.2
SS1G_00505	alpha-1,2-mannosidase	-	-	-	-	9.8	4.7
SS1G_04148	alpha-mannosidase	-	-	-	-	4.1	4
SS1G_02022	alpha-mannosidase	-	-	-	-	12.5	10.9
SS1G_04200	alpha-mannosidase	-	-	-	-	6	56.3
SS1G_01334	alpha-mannosidase	-	-	-	-	-	11.5
*2.8 Callose*							
SS1G_01422	1,3 (4)-beta-D-glucanase	2.2	-	-	-	-	-
SS1G_10048	1,3 (4)-beta-D-glucanase	-	2.1	-	-	2.1	-
*3. Protein Degradation*							
*3.1 Proteases*							
SS1G_10992	caspase domain-containing protease	2.1	-	-	-	-	7.1
SS1G_00862	cysteine protease (calpain family)	6.1	5.1	6.3	3.4	3.2	-
SS1G_09978	peptidase (family 41 protein)	3.2	-	3.2	2.9	-	6.4
SS1G_07836	(acid) non-aspartyl protease (ACP1)	-	-	-	-	-	40.4
SS1G_05329	aspartyl protease	12.2	-	-	-	-	4.6
SS1G_02870	aspartyl protease	2	2.6	3	3	2.7	3.3
SS1G_03181	aspartyl protease	-	-	-	-	3.9	16.6
SS1G_06534	serine protease (trypsin-like)	2.8	3	-	-	-	3.9
SS1G_12419	serine protease (subtilisin-like)	-	-	3	-	-	-
SS1G_07655	serine protease (subtilisin-like)	-	-	-	-	8.5	19.8
SS1G_02423	serine protease (subtilisin-like)	-	-	-	-	-	2.2
SS1G_03282	serine protease (subtilisin-like)	-	-	-	-	-	2.2
SS1G_07168	serine protease (subtilisin-like)	-	-	-	-	-	2.5
SS1G_12210	serine protease (subtilisin-like)	-	-	-	-	-	7.3
SS1G_05348	metalloprotease	-	-	-	-	-	2.6
SS1G_05349	metalloprotease	-	-	-	-	-	3.1
*3.2 Peptidases*							
SS1G_04565	cytosolic no-pecific dipeptidase	-	-	-	-	26	9.2
SS1G_10529	cytosolic no-pecific dipeptidase	-	-	-	-	-	2.2
SS1G_04140	dipeptidyl-peptidase	-	2.5	-	-	-	-
SS1G_03087	membrane dipeptidase	-	-	-	-	-	2.6
SS1G_03392	proline dipeptidase	-	-	-	-	4.5	-
SS1G_08920	proline dipeptidase	-	-	-	-	2.4	2
SS1G_04958	tripeptidyl-peptidase	-	-	-	-	5	22.9
SS1G_07268	tripeptidyl-peptidase	-	-	-	-	3.5	2.1
SS1G_13922	tripeptidyl peptidase	-	-	-	-	8.9	50.4
SS1G_09225	tripeptidyl peptidase	-	-	-	-	6.9	16.9
SS1G_09268	tripeptidyl-peptidase	-	-	-	-	5.9	3.4
SS1G_02857	tripeptidyl peptidase	-	-	-	-	6.4	2.5
SS1G_03518	tripeptidyl peptidase	-	-	-	-	-	6.1
SS1G_01236	tripeptidyl peptidase	-	-	-	-	-	9.7
SS1G_08558	prolyl aminopeptidase	2.5	4.3	4.4	3.4	2.4	2.2
SS1G_00617	prolyl aminopeptidase	-	-	-	9.5	4.9	5.2
SS1G_12775	prolyl aminopeptidase	-	-	-	-	-	3
SS1G_05449	carboxypeptidase	-	2.5	-	-	3.3	13.3
SS1G_12413	carboxypeptidase	-	-	-	-	3.5	4.2
SS1G_08855	carboxypeptidase	-	-	-	-	3.7	4.1
SS1G_03361	carboxypeptidase	-	-	-	-	4.2	21.1
SS1G_09475	carboxypeptidase	-	-	-	-	-	13.1
SS1G_13633	carboxypeptidase	-	-	-	-	-	7.6
SS1G_12499	carboxypeptidase	-	-	-	-	-	11.5
SS1G_04819	carboxypeptidase	-	-	-	-	-	5.5
*4. Other Hydrolytic Enzymes*							
SS1G_01113	metallo-dependent amidohydrolase	-	-	-	-	3.2	-
SS1G_09143	metallo-dependent amidohydrolase	-	-	-	-	-	6.1
SS1G_02141	alpha beta-hydrolase	4.6	-	-	-	-	-
SS1G_11096	alpha beta-hydrolase	-	-	-	2.1	5.7	2.6
SS1G_08093	alpha beta-hydrolase (epoxide hydrolase)	-	-	-	-	4.4	9.2
SS1G_04475	endo-alpha-1,4-polygalactosaminidase	3	-	-	-	-	3.8
SS1G_11842	sialidase	3.1	-	3.6	-	-	9.8
SS1G_01389	polysaccharide lyase family 7 protein	-	4.9	4.9	5.2	-	-
SS1G_01493	glycoside hydrolase family 3 protein	-	-	-	-	4.2	17
SS1G_09000	glycoside hydrolase family 5 protein	2.7	-	-	-	2.7	-
SS1G_02369	glycoside hydrolase family 12 protein	-	-	-	-	-	3.4
SS1G_04497	glycoside hydrolase family 16 protein	-	-	-	-	-	2
SS1G_09789	glycoside hydrolase family 16 protein	-	-	-	-	-	2.8
SS1G_06426	glycoside hydrolase family 43 protein	-	-	-	-	2.4	5.9
SS1G_07515	glycoside hydrolase family 43 protein	-	-	-	-	138	518
SS1G_07656	glycoside hydrolase family 61 protein	-	-	-	-	-	93.6
SS1G_09251	glycoside hydrolase family 61 protein	-	-	-	-	-	9.1
SS1G_12106	glycoside hydrolase family 76 protein	-	-	-	-	3	13
SS1G_12083	glycoside hydrolase family 115 protein	-	-	-	-	7.8	17
SS1G_04152	glycoside hydrolase family 125 protein	-	2.2	-	-	6	12.5
SS1G_12917	glycoside hydrolase family 128 protein	-	-	-	-	2.3	17.3

^a^Annotation based on the presence of conserved PFAM domains and BLAST reports

^b^Fold change relative to 0 h post inoculation (hpi). (-) No significant change in expression

More information about the genes can be found in Additional file 3: Table S3

##### 1.1 Cutinases/lipases

The first barrier to pathogen invasion is the plant cuticle, a composite layer of C:16 and C:18 fatty acids and their derivatives that form cutin and surface waxes [50]. Among the genes from the CE family, the gene encoding the cutinase, SsCuta (SS1G_07661), was up-regulated during the early stages of infection (from 1-24 hpi). The induction of *SsCuta* soon after contact with the leaf surface in the current study agrees with the previous report showing that it was induced upon contact of mycelia with hard surfaces [4] and supports the predicted role of this enzyme in degrading plant cuticle. Additionally, it is not surprising that expression of this gene declined after 24 hpi since host penetration has already been achieved by this time. In addition to *SsCuta*, three other genes from the lipid degradation group (Table 1), SS1G_09557, SS1G_01953 and SS1G_11930, were also induced during the early stages of infection. The similarity between the expression patterns of these genes and that of *SsCuta*, as well as their potential lipolytic enzymatic activity, suggest that these enzymes may also be involved in host penetration. Evidence that lipase acts as virulence factor in fungal phytopathogens was observed in *Botrytis cinerea* (*Lip1*) [51]. The secreted lipase in *Fusarium graminearum* encoded by *FgL1* is also a virulence factor contributing to the infection of cereals [52]. Genes encoding other lipases and members of the CE family, such as the cutinases (SS1G_13386 and SS1G_12907) and an extracellular lipase (SS1G_14146), were significantly up-regulated at 24 and 48 hpi (Table 1).

##### 1.2 Plant cell wall degrading enzymes

Once the cuticle has been breached, the pathogen must establish within the host and then proceed to ramify through host tissues. The production of enzymes that degrade plant cell wall components physically allows this to occur, while providing nutrients to drive the infection process [53]. The primary plant cell wall is composed mainly of cellulose, hemicellulose and pectin, along with structural glycoproteins (e.g. hydroxyproline-rich extensins) and phenolic esters (e.g. ferulic and coumaric acid). The secondary cell wall consists mostly of lignin, a highly cross-linked phenolic macromolecule.

The GH28 subfamily contains the polygalacturonases (PGs), enzymes that degrade cell wall pectin. Previously, four genes encoding endo-PGs (*SsPG1*, *SsPG3*, *SsPG5*, and *SsPG6*) and two genes encoding exo-PGs (*SsXPG1* and *SsXPG2*) were found to be expressed during *S. sclerotiorum* infection of *B. napus* [23]. In the current study, the genes encoding SsPG1 (SS1G_10167) and SsPG3 (SS1G_10698) were up-regulated at 24-48 hpi and 48 hpi with expression levels 3.7- 64.6 and 5.5-fold greater than the inoculum, respectively. The two exo-PGs genes, *SsXPG1* (SS1G_04207) and *SsXPG2* (SS1G_02553), were up-regulated at 24-48 hpi and 48 hpi with expression levels 14.6-168.3 and 22.6- fold greater than the inoculum, respectively. While the main pectin backbone is a homopolymer of α-(1 → 4)-linked D-galacturonic acid residues, branched and unbranched side chains are appended to it that contain several different types of sugars. Most of the genes encoding these ancillary pectin-degrading enzymes were highly expressed at 24 and 48 hpi in concert with the endo-PGs genes *SsPG1* and *SsPG3*, except for SS1G_05832 (exo-PG), SS1G_04095 (rhamnogalacturanan acetylhydrolase) and SS1G_08229 (rhamnogalacturonase) which were up-regulated at 1-3 hpi, 1-24 hpi and 1-12 hpi, respectively.

Li et al. [23] reported that *SsPG1* expression could be induced by contact with hard surfaces, while Bashi et al. [4] reported that *SsPG1*, but not *SsPG2*, was moderately induced by contact with *B. napus* leaves and that *SsPG1* expression was restricted to the expanding margin of the lesion. They suggested that since *SsPG1* expression was also induced by carbon starvation and repressed by galacturonic acid that it may be involved in both early penetration events and lesion expansion. During *Phaseolus vulgaris* infection, *SsPG1* is induced during the later stages of the interaction (48-72 hpi), *SsPG*3 is up-regulated earlier at 12 hpi, while *SsPG6* exhibits a bimodal pattern with peaks of expression at 6 and 48 hpi [40]. SsPG3 and SsPG6 are also potent inducers of light-dependent necrotic reactions [54]. Similarly, *B. cinerea BcPG1* and *BcPG2* exhibit strong necrosis-inducing activity [55] and deletion of either gene reduces *B. cinerea* virulence [55, 56]. The induction of SsPG or orthologous genes well after host penetration and their ability to cause tissue necrosis suggests that the primary role of these enzymes is in lesion expansion and movement of the pathogen through the host tissues.

Many of the up-regulated GH and CE family members reported in this study also have a putative role in the degradation of hemicellulose and cellulose. This was similar to the previous results obtained for up-regulated GH genes in *S. homoeocarpa* [41]. Cellulose is a homopolymer of beta-(1, 4)-linked D-glucose and is sequentially hydrolyzed into its component glucose by enzymes including cellulases (endo-1,4-glucanases), cellobiosidases (exo-glucanases) and beta-glucosidases. Numerous genes encoding putative cellulases were up-regulated at some point during the infection, mostly at the later stages, with SS1G_09821 and SS1G_03041, up-regulated 144 and 292 fold at 48 hpi (Table 1). Genes encoding putative exo-glucanases and beta-glucosidases followed a similar pattern of expression with most being up-regulated at the later stages and only a few during the earlier stages of the infection. In higher plants, hemicellulose comprises approximately 20% of the total biomass. Unlike the more homogenous cellulose, hemicellulose is composed not only of glucose, but of other sugars such as xylose, mannose, galactose, rhamnose, and arabinose. As such, its deconstruction requires a more complicated bevy of enzymatic reactions. Similar to the genes encoding cellulose-degrading enzymes, genes encoding putative hemicellulose degrading enzymes were also up-regulated later in the infection. The exceptions were genes encoding enzymes involved in the release of xylose from xylan (beta-1,4-linked xylose), namely SS1G_12191, SS1G_05140 and SS1G_08104, which were first induced at the earlier stages. Two other genes encoding xylanases (SS1G_10092 and SS1G_03618) were among the most highly induced genes found in this study with levels of expression 155 and 451 fold higher than that of the inoculum at 48 hpi. These patterns may attest to the abundance of this sugar in the plant cell wall and/or its significance to *S. sclerotiorum* nutrition. Interestingly, SS1G_10092 is likely orthologous to the gene encoding *B. cinerea* xylanase11A (90% amino acid identity) which induces a strong necrotic reaction and is required for virulence [57]. Both proteins share a 30 amino acid region associated with necrotizing activity [57]. In the *S. sclerotiorum*– *P. vulgaris* interaction, SS1G_01493 (beta-xylosidase) was up-regulated during the early stages before the emergence of visible necrotic symptoms on the stem, whereas genes encoding cellulose-degrading enzymes, SS1G_13255 (beta-1,4-glucanase) and SS1G_07146 (cellobiohydrolase), were induced during the later stages of infection coinciding with the formation of visible stem lesions [40]. In *B. cinerea*, the expression patterns of genes encoding xyloglucan-degrading enzymes was found to be vastly different dependent upon the host plant [58].

Two genes encoding other hemicellulose-degrading enzymes with alpha-L-arabinofuranosidase activity, SS1G_02462 and SS1G_03602, were also up-regulated at 24-48 hpi. These enzymes target the L-arabinofuranose residues of hemicellulose with pectin side chains [59]. Alpha-L-arabinofuranosidase was first reported in *S. sclerotiorum* by Yajima and Kav [42]. An earlier study on *Sclerotinia fructigena* suggested that there was significant correlation between the quantity of alpha-L-arabinofuranosidase and virulence of this fungus through its contribution to disease initiation or fungal proliferation [60].

Arabinogalactans are structurally complicated branched galactans with arabinose side chains and can be found as either beta-1,4-galactans linked to rhamnogalacturonan I in pectin, or as beta-1,4-galactans associated with proteins [61]. As with the other cell wall degrading enzymes, the majority of the genes encoding putative arabinogalactan-degrading enzymes were induced later in the infection (24-48 hpi) (Table 1). However, a gene encoding an arabinogalactan endo-beta-galactosidase (SS1G_01216) and another encoding a beta-galactosidase (SS1G_10842) were already induced at 1 hpi.

Mannans are polymers of mannose. Those with β (1–4) linkages are typical of plant storage polysaccharides, while mannans with α (1–6) linked backbone and α (1–2) and α (1–3) linked branches are often associated with glycoproteins. As noted above, glycoproteins are a significant cell wall component and several genes encoding mannosidases capable of hydrolyzing these chemical bonds were up-regulated during the later stages of the infection (Table 1).

Lignin provides additional structure and rigidity to the plant cell wall and increased lignification is often a consequence of imposed biotic and abiotic stresses [62]. Three genes encoding extracellular dihydrogeodin oxidases were up-regulated at the mid to later stages of the infection (Table 1). All contain three multicopper oxidase domains which are often associated with enzymes, such as laccases, that oxidize phenolic compounds. Laccases are involved in the disassembly of lignin [63], though some may detoxify phenolic secondary metabolites as discussed below.

Collectively, the plethora of CAZymes expressed by *Sclerotinia* species allow this group of pathogens to break down most host polysaccharides to efficiently access nutrients from a wide variety of hosts. The expression patterns of the CAZyme genes in the current study, which were mostly expressed at later stages of infection (24-48 hpi), support the hypothesis that they are primarily involved in tissue maceration. Factors such as host plant species, the type of tissues being colonized and the environment also influence the expression of different CAZyme genes. In *B. cinerea*, the expression of PG genes, in particular *BcPG1* and *BcPG2*, was markedly different on different hosts or on the same host at different temperatures [58]. This same phenomenon was observed with genes encoding various pectin lyases, pectate lyases and pectin methylesterases [58]. The large number of genes encoding CAZymes and their different patterns of expression strongly indicate that they make an important contribution to pathogenesis and host range through adaptation to various environmental and host factors.

##### 1.3 Proteases

Although CAZymes have captured most of the attention as the main group of hydrolytic enzymes involved in pathogenesis, there are other groups of hydrolytic enzymes which also play crucial roles. Many genes encoding endo-proteases, as well as mono-, di- and tri-peptidyl peptidases were up-regulated during infection (Table 1). The *in planta* expression of the *acp1* (non-aspartyl acid protease, SS1G_07836) was recorded during *S. sclerotiorum* infection of sunflower cotyledons with the peak expression level at 24-56 hpi [21]. *Acp1* was also up-regulated in the current study with 40-fold greater expression than the inoculum at 48 hpi. The expression of this gene only at the necrotic stage is in support of the previous study. During infection of *P. vulgaris*, *acp1* is first induced during the very early stages of the infection and then again at the later stages [40]. The expression of *acp1* is regulated by several environmental factors including glucose and nitrogen starvation and acidification. The *PacC* transcription factor is involved in the regulation of *acp1* expression under acidic conditions [21].

A gene encoding an aspartyl protease (*aspS,* SS1G_03629) from *S. sclerotiorum* was previously reported as being induced at the very early stages of the infection and was involved in decomposition of host defense proteins [22]. An aspartyl protease was also reported as a cell death-inducing factor secreted by *S. sclerotiorum* and *B. cinerea* [64]. Although the *aspS* gene was not up-regulated in the current study, genes encoding proteases similar to aspS (SS1G_05329 and SS1G_02870), were up-regulated at the very early stages of the infection in support of the previous studies. However, a gene encoding another aspartyl protease*,* SS1G_03181, was also detected in the *S. sclerotiorum*– *P. vulgaris* interaction with increased expression at the spreading necrosis stage [40]. SS1G_03181 was up-regulated at 24-48 hpi in the current study which is in agreement with these earlier findings.

Most of the genes encoding subtilisin-like serine proteases (SS1G_07655, SS1G_02423, SS1G_03282, SS1G_12210 and SS1G_07168) were up-regulated at the later stages of the infection (24-48 hpi) (Table 1). Subtilisins were proposed to play a role in penetration and colonization because of their ability to degrade plant cell wall glycoproteins or pathogenesis-related proteins [65]; however, the expression profiles of genes encoding these enzymes in the current study suggest that they are also involved in events occurring at the necrotic stage. Genes encoding enzymes with metalloprotease activities (SS1G_05348 and SS1G_05349) were also up-regulated at 48 hpi. These proteases play a role in degrading plant materials for nitrogen utilization [66]. In accordance with this notion, the majority of the genes encoding exo-peptidases, which complete the hydrolysis of peptides generated by endo-proteases into their component amino acids, were expressed at the later stages of the infection similar to the genes encoding serine and metalloproteases (Table 1).

The current study revealed one calpain family cysteine protease gene (SS1G_00862) that was induced at 1 hpi and continued to be expressed during the middle stages of the infection out to 24 hpi. A caspase domain-containing cysteine protease (SS1G_10992) was also up-regulated at 1 hpi and was detected again at 48 hpi at a higher expression level (7.1-fold greater than the inoculum). According to previous studies, these types of endo-peptidases contribute to programmed cell death (PCD) processes (reviewed by [67]). Among these, cysteine proteases, specifically the caspases, have a key role in PCD, more commonly apoptosis (reviewed by [67]). In addition, cysteine proteases along with other proteolytic systems such as calpain, 26S proteasome, granzyme B, cathepsin D and matrix metalloproteinases also have a role during PCD processes [68]. There is a report suggesting that victorin, a toxin produced by *Cochliobolus victoriae*, induces proteolytic cleavage of the Rubisco large subunit (LSU) through activation of a host cysteine protease [69]. Considering these previous studies, understanding the precise role of these genes in the pathogenesis of *S. sclerotiorum* warrants further investigation as they may be involved in processes linked to phase transitions during the infection [11].

Proteases are important for *S. sclerotiorum* nutrition as protein forms about 10% of host cell protoplasm [70]. Numerous proteases were also captured in the *S. homoeocarpa* transcriptome, but these were mostly serine endo-proteases [41]. In the current study, the up-regulated endo-protease genes encoded enzymes with a much broader range of catalytic mechanisms. Several genes encoding putative aspartyl (active at acidic pH) and cysteine (active at acidic to neutral pH) proteases were up-regulated very early in the infection, while most of the serine proteases (active at high pH) and two genes encoding metalloproteases were up-regulated at the later stages. It is possible that the coordinated and systematic deployment of proteolytic enzymes with different catalytic mechanisms reflects the lesion environment, the pathogen’s nutritional requirements and interactions with host defense systems at different stages of the infection.

#### 2. Secondary metabolite biosynthesis

Several genes encoding enzymes involved in the biosynthesis of secondary metabolites were up-regulated in the current study (Table 2). These included key enzymes associated with pathways for production of toxic compounds, including polyketide synthase (PKS), nonribosomal peptide synthase (NRPS), hybrid PKS/NRPS, and chalcone synthase (CHS). These fungal toxins interfere with host cell functions to suppress plant defense and/or enhance development of disease symptoms [71]. Of the various types of phytotoxic metabolites produced by *B. cinerea*, botrydial has been most intensively studied [72]. *BcBOT1* is part of the botrydial biosynthesis pathway and encodes a cytochrome P450 monooxygenase [73]. Cytochrome P450 enzymes are also involved in the aflatoxin biosynthetic pathways [74]. SS1G_09638 and SS1G_11247 have some similarity to *StcL* gene, which is involved in aflatoxin biosynthesis in *Aspergillus nidulans*. These genes were expressed, but not up-regulated compared to the inoculum in the current study, while SS1G_13923 was up-regulated at 1 to 6 hpi (Table 3). It should be noted that while cytochrome P450 enzymes are involved in the production of secondary metabolites and mycotoxins, some members are also involved in the detoxification of host metabolites in different fungi [75].

**Table 2:** Description and expression of up-regulated genes involved in secondary metabolite synthesis

Gene ID	Description^a^	Expression level (hpi)^b^					
		1	3	6	12	24	48
*1. Polyketides*							
SS1G_02356	polyketide synthase (PKS1)^c^	-	-	-	5.1	7.5	5
SS1G_05681	polyketide synthase (PKS4)^c^	-	-	3.4	2.5	-	-
SS1G_09237	polyketide synthase (PKS6)^c^	-	-	-	-	-	34.3
SS1G_01997	polyketide synthase (PKS8)^c^	3.1	4.9	6.4	4.5	-	-
SS1G_11789	polyketide synthase (PKS12)^c^	-	2.3	-	-	-	2.4
SS1G_11404	polyketide synthase (PKS14)^c^	2.8	2.1	2.9	4.7	4.7	9.2
SS1G_04125	polyketide synthase (PKS16)^c^	2.7	3	-	-	3.5	-
SS1G_05787	polyketide synthase (PKS18)^c^	-	-	-	-	3.5	3
SS1G_03591	polyketide synthase (PKS21)^c^	-	-	2.5	-	2.2	-
SS1G_04127	polyketide synthase	2.1	-	-	-	2.9	-
SS1G_10474	polyketide synthase	4	-	-	-	5.2	-
SS1G_05788	polyketide synthase	-	-	-	2.5	4.9	3.5
SS1G_11352	DBSA oxidoreductase (FmE)	4.4	2.8	3.3	-	-	-
SS1G_09239	FAD-dependent monooxygenase (similar to BcBOA8)	-	-	-	-	10.9	17.6
SS1G_02592	zinc-binding oxidoreductase (ToxD)	4	-	-	4.2	5.8	17.8
SS1G_02338	chalcone and stilbene synthase (CHS1)	-	-	-	-	2.1	-
*2. Non-ribosomal Peptides*							
SS1G_08561	non-ribosomal peptide synthase (NRPS1)^c^	-	-	-	4.7	8.9	32.5
SS1G_03693	siderophore peptide synthase (NRPS2)^c^	-	2.2	2.3	-	4	3.1
SS1G_06185	siderophore peptide synthase (NRPS3)^c^	3.6	8.2	8.8	9.3	4.7	-
SS1G_10563	non-ribosomal peptide synthase (NRPS5)^c^	-	-	-	-	2.3	-
SS1G_12986	NRPS-like enzyme	-	6.3	5.2	4.3	5.8	7
SS1G_09846	NRPS-like enzyme	2.9	2.2	2.6	-	2.1	-
SS1G_03440	NRPS-like enzyme	-	-	-	3.5	4	-
SS1G_00726	NRPS-like enzyme	-	-	-	18.8	-	-
SS1G_01217	NRPS-like enzyme	-	-	-	5.4	10.5	7.7
*3. Other*							
SS1G_09177	siroheme synthase	10.9	8.7	8.3	5.7	-	-

^a^Annotation based on the presence of conserved PFAM domains and BLAST reports

^b^Fold change relative to 0 h post inoculation (hpi). (-) No significant change in expression

^c^Gene abbreviations used by Amselem et al., 2011 [38]

More information about the genes can be found in Additional file 3: Table S3

**Table 3:** Description and expression of up-regulated genes encoding cytochrome p450 enzymes

Gene ID	Description^a^	Expression level (hpi)^b^					
		1	3	6	12	24	48
SS1G_02340	cytochrome p450 (pistatin demethylase)	3.0	5.4	6.7	5.1	-	-
SS1G_04805	CYP51 (eburicol 14 alpha-demethylase)	3.4	3.6	2.4	2.2	-	-
SS1G_00406	CYP55a3 (nitric oxide reductase)	2.1	2.5	2.1	-	-	-
SS1G_00623	cytochrome p450	2.3	-	-	-	-	2.5
SS1G_13470	cytochrome P450 (alkane hydroxylase)	2.2	-	-	-	-	-
SS1G_05491	cytochrome p450	-	86	271	495	1067	5372
SS1G_13909	cytochrome P450	-	2.1	2.2	-	-	-
SS1G_05384	cytochrome p450	-	2.6	-	2.4	-	-
SS1G_02363	cytochrome p450 (trichodiene oxygenase)	-	-	-	4.2	3.3	3.9
SS1G_04088	cytochrome p450 (oxidoreductase)	-	-	-	3.5	7.5	18.7
SS1G_11553	cytochrome p450	-	-	-	2.0	2.3	2.9
SS1G_03436	cytochrome p450 (benzoate 4-monooxygenase)	-	-	-	4.0	2.6	-
SS1G_05490	cytochrome p450 (benzoate 4-monooxygenase)	-	-	-	-	4.6	8.6
SS1G_01843	cytochrome p450 (benzoate 4-hydroxylase)	-	-	-	-	5.5	3.0
SS1G_13957	cytochrome p450	-	-	-	-	14.9	20.4
SS1G_02157	cytochrome p450	-	-	-	-	3.6	9.9
SS1G_04780	cytochrome p450	-	-	-	-	2.2	2.8
SS1G_06101	cytochrome p450	-	-	-	-	4.9	12.7
SS1G_10037	cytochrome p450	-	-	-	-	8.6	9.0
SS1G_11430	cytochrome p450 (alkane hydroxylase)	-	-	-	-	2.1	4.7
SS1G_11697	cytochrome p450	-	-	-	-	2.4	2.5
SS1G_14168	cytochrome p450	-	-	-	-	2.1	6.0
SS1G_11768	cytochrome p450	-	-	-	-	5.3	-
SS1G_01006	cytochrome p450	-	-	-	-	-	3.5
SS1G_08136	cytochrome p450	-	-	-	-	-	7.0
SS1G_14163	cytochrome p450	-	-	-	-	-	3.1
SS1G_08677	cytochrome P450 (monooxygenase)	-	-	-	-	-	2.8

^a^Annotation based on the presence of conserved PFAM domains and BLAST reports

^b^Fold change relative to 0 h post inoculation (hpi). (-) No significant change in expression

More information about the genes can be found in Additional file 3: Table S3

Polyketides are a structurally diverse group of secondary metabolites derived from the decarboxylative condensation of malonyl-CoA and include many mycotoxins. In *B. cinerea*, two genes encoding PKS, *BcPKS6* and *BcPKS9*, that act in concert to synthesize the phytotoxin botcinic acid, are up-regulated during infection of tomato leaves [76]. Transcripts from the *S. sclerotiorum* ortholog of *PKS6* (SS1G_09237) were detected at 48 hpi, while *PKS9* (SS1G_09240) was expressed, but not up-regulated in this study. This agrees with the findings of Pedras and Ahiahonu [77] regarding the absence of botcinic acid in *S. sclerotiorum* as both enzymes are required for its synthesis. Both botrydial and botcinic acid toxins contribute to the virulence of *B. cinerea* [76]. SS1G_02592 encodes a protein similar to ToxD which is involved in the synthesis of the polyketide lovastatin in *Aspergillus terreus* [78]. It was initially up-regulated within 1 hpi with expression peaking at 48 hpi.

Among the genes similarly induced during the infection by *B. cinerea* and *S. sclerotiorum* are those conserved in ascomycetes and involved in the biosynthesis of melanin (*PKS13*), coprogen (*NRPS6*) and intracellular siderophores (*NRPS2*, *NRPS3*) [38]. *PKS13* (SS1G_13322) and *NRPS6* (SS1G_04250) were expressed, but not up-regulated in the current study, while homologues of the genes responsible for siderophore biosynthesis *NRPS2* and *NRPS3* (SS1G_03693T0 and SS1G_06185T0) were up-regulated at 3-48 and 1-24 hpi, respectively. Siderophores scavenge iron and are important virulence factors for many pathogens, including phytopathogenic fungi such as *Cochliobolus heterostrophus* [79] and *Aspergillus fumigatus* [80]. Iron-containing cofactors, such as heme and siroheme are required for the catalytic activity of enzymes associated with nitrogen and sulfur assimilation, as well as xenobiotic detoxification [81]. In the current study, a gene encoding siroheme synthase (SS1G_09177), also known as S-adenosyl-L-methionine:uroporphyrinogen III methyltransferase, was sharply induced during the earliest stages of the infection (1-12 hpi) attesting to the importance of iron sequestration and metabolism.

In addition to key enzymes involved in secondary metabolite biosynthesis, transporters are also required to deploy or secrete secondary metabolites (Table 4). For example, HC-toxin is a virulence factor of *Cochliobolus carbonum* on maize and is synthesized by a NRPS named HTS1 [82]. It is exported from the cell by two major facilitator superfamily (MFS) transporters, TOXA and TOXB [83]. Three genes encoding transporters with similarity to TOXA proteins were up-regulated in the current study. The proteins encoded by SS1G_09759, SS1G_00919 and SS1G_06662 exhibited 51% similarity to putative HC-toxin efflux carrier TOXA from *Aspergillus lentulus*, 61% to the HC-toxin efflux carrier TOXA from *Glarea lozoyensis* and 84% to the MFS toxin efflux pump protein of *B. cinerea*, respectively. It might therefore be inferred that these transporters are involved in the efflux of toxins into the host plant during *S. sclerotiorum* infection, but characterization of their precise substrate specificities requires further study.

**Table 4:** Description and expression of up-regulated genes encoding transporters

Gene ID	Description^a^	Expression level (hpi)^b^					
		1	3	6	12	24	48
*1. ABC transporter superfamily* * 1.1 Pleiotropic Drug Resistance*							
SS1G_06715	Pleiotropic Drug Resistance (PDR)	2.0	-	-	-	-	-
SS1G_13659	pleiotropic drug resistance-like (PDR)	-	-	-	-	3.1	13.5
SS1G_04483	pleiotropic drug resistance-like (PDR)	-	-	-	-	-	4.6
*1.2 Multi Drug Resistance*							
SS1G_04756	ABC multidrug transporter	3.4	7.9	6.5	3.7	2.4	-
SS1G_07287	ABC multidrug transporter	3.5	3.2	2.6	-	-	2.2
SS1G_03324	ABC multidrug transporter	3.5	4.8	4.4	-	-	-
SS1G_13112	ABC multidrug transporter	-	4.1	6.2	3.9	-	-
SS1G_02000	ABC multidrug transporter	-	-	3.9	3.2	-	-
SS1G_06062	ABC multidrug transporter	-	-	-	-	2.4	2.8
*1.3 Other families*							
SS1G_04757	mitochondrial ATP-binding cassette protein involved in iron homeostasis	-	6.5	5.1	3.4	-	-
SS1G_07963	ABC transporter with iron-containing redox enzyme with death domains	-	-	-	-	4.0	8.5
SS1G_12070	ABC lipid transporter	-	-	-	-	-	2.6
SS1G_10747	ABC peroxysomal fatty Acyl CoA transporter	-	-	-	-	-	3.2
SS1G_05904	ABC peroxysomal fatty Acyl CoA transporter	-	-	-	-	-	2.0
*2. MFS transporter superfamily*							
*2.1 Efflux pumps and drug resistance*							
SS1G_10155	MFS drug efflux and drug resistance	10.1	5.5	3.7	-	-	-
SS1G_11948	MFS drug efflux and drug resistance	2.8	6.7	5.5	-	-	-
SS1G_00919	MFS gliotoxin efflux transporter	-	2.7	3.0	-	-	-
SS1G_09759	MFS aflatoxin efflux transporter	-	8.0	-	-	-	-
SS1G_05556	MFS drug efflux and drug resistance	-	-	2.4	-	-	-
SS1G_06662	MFS toxin efflux pump	-	-	3.0	-	-	-
SS1G_05572	MFS drug resistance transporter	11.4	2.9	-	-	-	-
SS1G_02358	MFS drug resistance transporter	-	-	-	4.1	3.7	4.3
SS1G_12101	MFS drug resistance transporter	-	-	-	-	2.4	-
SS1G_02394	MFS drug resistance transporter	-	-	-	-	-	4.6
SS1G_05756	MFS drug resistance transporter	-	-	-	-	-	15.2
SS1G_10566	MFS drug resistance transporter	-	-	-	-	-	5.5
SS1G_05556	MFS drug resistance transporter	-	-	-	-	-	2.0
SS1G_05095	MFS fungal trichothecene efflux pump (TRI12)	-	-	-	-	-	7.1
SS1G_05145	MFS fungal trichothecene efflux pump (TRI12)	-	-	-	-	5.0	-
SS1G_13024	MFS drug resistance transporter	-	-	-	-	-	3.6
SS1G_11919	MFS multidrug transporter	-	2.7	2.3	-	-	-
SS1G_02623	MFS multidrug transporter	-	-	-	-	-	4.7
SS1G_08554	MFS multidrug transporter	-	-	-	-	-	2.9
SS1G_10279	MFS multidrug transporter	-	-	-	-	-	2.1
SS1G_09892	MFS multidrug transporter	-	-	-	-	-	10.2
SS1G_02931	MFS multidrug transporter	-	-	-	-	-	2.8
SS1G_02048	spermidine drug resistance transporter	2.1	-	-	-	-	3.2
*2.2 Sugar transporters*							
SS1G_04841	hexose transporter	4.1	2.9	-	-	-	-
SS1G_13734	hexose transporter HXT13	-	2.1	-	-	-	-
SS1G_04273	hexose transporter	-	-	-	-	6.1	15.8
SS1G_12412	hexose transporter	-	-	2.1	-	2.5	2.4
SS1G_09710	MFS sugar transporter	-	-	2.7	2.6	4.8	6.5
SS1G_06023	MFS monosaccharide transporter	6.9	6.7	7.0	4.9	-	-
SS1G_10125	MFS monosaccharide transporter	2.1	2.4	-	-	-	-
SS1G_06620	MFS monosaccharide transporter	-	-	-	-	2.4	-
SS1G_07618	MFS monosaccharide transporter	-	-	-	-	-	2.1
SS1G_08794	MFS sucrose transporter	-	-	2.6	-	-	-
SS1G_04208	MFS sugar transporter	-	-	-	4.0	36.7	49.2
SS1G_10413	MFS sugar transporter	-	-	-	2.3	4.6	4.8
SS1G_06751	MFS sugar transporter	-	-	-	-	2.0	-
SS1G_08117	MFS sugar transporter	-	-	-	-	2.8	-
SS1G_11591	MFS sugar transporter	-	-	-	-	10.7	9.4
SS1G_01523	MFS sugar transporter	-	-	-	-	13.1	12.5
SS1G_00928	MFS sugar transporter	-	-	-	-	7.2	10.6
SS1G_08467	MFS sugar transporter	-	-	-	-	2.9	2.5
SS1G_07132	MFS sugar transporter	-	-	-	-	8.3	6.3
SS1G_01759	MFS sugar transporter	-	-	-	-	-	2.8
SS1G_14316	MFS sugar transporter	-	-	-	-	-	6.1
SS1G_06402	MFS sugar transporter	-	-	-	-	-	16.2
SS1G_01302	MFS sugar transporter	-	-	-	-	-	47.6
SS1G_03579	MFS sugar transporter	-	-	-	-	-	4.1
SS1G_05006	MFS sugar transporter	-	-	-	-	-	11.8
SS1G_08982	MFS quinate transporter	-	-	-	-	18.9	26.1
SS1G_09293	MFS quinate transporter	-	-	-	-	3.5	2.9
SS1G_08981	MFS quinate transporter	-	-	-	-	49.3	38.4
SS1G_00139	MFS carbohydrate transporter	-	3.1	2.2	2.0	-	-
SS1G_04149	MFS carbohydrate transporter	-	3.2	2.5	-	-	8.2
SS1G_07210	MFS carbohydrate transporter	-	-	-	-	2.5	2.3
SS1G_08328	MFS carbohydrate transporter	-	-	-	-	5.8	4.1
SS1G_13671	MFS carbohydrate transporter	-	-	-	-	2.4	-
SS1G_14028	MFS carbohydrate transporter	-	-	-	-	2.1	2.0
SS1G_13819	MFS carbohydrate transporter	-	-	-	-	-	8.1
SS1G_11142	MFS monocarboxylate transporter	-	-	-	-	3.6	-
SS1G_01656	MFS monocarboxylate transporter	-	-	-	-	-	2.2
SS1G_07861	MFS galactonate transporter	2.8	-	-	-	-	-
SS1G_09368	MFS galactonate transporter	-	-	3.6	5.8	8.3	9.0
SS1G_09803	MFS galactonate transporter	-	-	-	4.2	14.9	13.1
SS1G_01055	MFS galactonate transporter	-	-	-	-	3.8	-
SS1G_10065	MFS galactonate transporter	-	-	-	-	73.2	487.6
SS1G_11224	MFS galactonate transporter	-	-	-	-	3.3	2.2
SS1G_11609	MFS galactonate transporter	-	-	-	-	3.0	13.0
SS1G_12649	MFS galactonate transporter	-	-	-	-	6.9	2.9
SS1G_12698	MFS galactonate transporter	-	-	-	-	4.0	5.7
SS1G_05090	MFS galactonate transporter	-	-	-	-	7.2	5.7
SS1G_10896	MFS alpha glucoside:h + symporter	-	-	-	-	4.4	2.3
SS1G_13094	MFS alpha glucoside:h + symporter	-	-	-	-	5.0	18.6
SS1G_03673	4-hydroxyphenylacetate permease and sugar transporter	-	-	-	-	-	5.3
SS1G_04849	MFS-fucose permease	-	-	-	-	-	3.1
SS1G_02117	lactose permease	-	-	-	2.8	23	31.3
SS1G_08398	maltose permease	-	4.2	-	-	-	3.8
SS1G_07922	maltose permease	-	-	-	-	2.2	-
SS1G_12954	maltose permease	-	-	-	-	-	8.2
*2.3 Oligopeptides*							
SS1G_02676	oligopeptide transporter	2.0	-	-	-	4.0	5.2
SS1G_09296	oligopeptide transporter	-	-	-	-	3.3	3.1
SS1G_09297	oligopeptide transporter	-	-	-	-	-	2.8
SS1G_10121	MFS peptide transporter	5.9	7.4	5.7	3.0	-	-
SS1G_07522	MFS peptide transporter	-	-	2.1	-	-	4.1
*2.4 others*							
SS1G_08628	MFS transporter	5.9	6.6	5.6	3.0	2.4	-
SS1G_04769	MFS transporter	-	-	-	-	-	7.3
SS1G_13941	MFS general substrate transporter	-	3.0	-	-	-	-
SS1G_13194	MFS general substrate transporter	-	-	-	-	2.2	-
SS1G_07517	MFS vacuole effluxer Atg22 transporter	-	3.5	3.5	-	2.7	2.4
SS1G_04978	MFS sulfate transporter	-	-	-	-	2.2	-
SS1G_12690	MFS nitrite transporter	-	7.0	12.2	10.7	-	-
SS1G_02906	MFS nicotinic acid transporter	-	-	-	-	3.8	2.1
*3. Amino acid transporters*							
SS1G_08387	amino acid transporter	-	-	-	3.0	10.4	9.5
SS1G_11233	amino acid transporter	-	-	-	-	2.7	-
SS1G_01802	amino acid transporter	-	-	-	-	-	3.9
SS1G_09839	amino acid transporter	-	-	-	-	-	13.3
SS1G_14102	choline, amino acid permease transport	-	-	-	2.3	-	-
SS1G_05293	choline, amino acid permease transport	-	-	-	-	-	5.0
SS1G_06535	choline, amino acid permease transport	-	-	-	-	-	5.1
SS1G_06536	choline, amino acid permease transport	-	-	-	-	-	3.1
SS1G_04884	ammonium transporter	-	-	-	-	6.8	-
SS1G_11781	arginine permease	2.2	-	-	-	-	-
SS1G_11563	amino acid permease	3.2	4.7	4.9	5.5	-	-
SS1G_02549	amino acid permease	3.1	4.2	3.9	2.2	-	-
SS1G_14381	amino acid permease	2.6	4.6	3.8	3.3	-	-
SS1G_13916	amino acid permease	9.1	11.5	8.6	6.9	2.3	-
SS1G_10633	amino acid permease	-	2.1	2.7	2.1	2.4	4.9
SS1G_03403	amino acid permease	-	-	-	-	3	-
SS1G_11780	amino acid permease	-	-	-	-	-	2.9
SS1G_06841	amino acid permease	-	-	-	-	-	2.7
*Others*							
SS1G_03654	formate nitrate transporter	3.0	3.2	3.1	3.4	4.0	3.9
SS1G_09621	mitochondrial phosphate carrier protein	3.8	5.0	4.4	3.3	-	3.0
SS1G_01720	C4-dicarboxylate transporter	20.2	19.0	18.8	8.5	-	-
SS1G_02619	C4-dicarboxylate transporter	2.2	2.7	2.6	-	-	-
SS1G_12337	calcium proton exchanger	2.5	3	2.2	-	-	2.6
SS1G_02802	putative Mg2+ transporter	2.6	2.8	2.4	-	-	3.8
SS1G_02548	UDP-N-acetylglucosamine transporter	2.4	2.8	2.8	-	-	-
SS1G_11564	lysosomal cystine transporter	2.1	3.0	2.5	-	-	-
SS1G_06298	cation efflux family transporter	3.1	4.0	3.5	-	-	-
SS1G_09822	monocarboxylate permease-like protein	2.1	2.5	-	-	-	2.7
SS1G_06910	vacuolar iron transporter	2.7	-	2.3	-	3.1	4.0
SS1G_03019	carnitine mitochondrial carrier protein	2.4	-	2.6	-	-	3.4
SS1G_08146	AGZA family xanthine/uracil permease	3	-	-	-	-	-
SS1G_10280	polyamine transport protein	-	2.7	2.1	-	-	-
SS1G_04686	phosphate/sulfate permease	-	4.2	-	-	3.3	-
SS1G_12991	cation, potassium transporter	-	-	2.4	-	-	-
SS1G_08795	succinate fumarate, mitochondrial transporter	-	-	5.4	-	-	8.0
SS1G_06006	purine-cytosine permease	-	-	-	6.4	3.7	-
SS1G_03305	solute carrier protein	-	-	-	3.0	-	-
SS1G_11712	cation, magnesium transporter	-	-	-	-	2.4	-
SS1G_07645	cation, potassium transporter	-	-	-	-	2.2	2.5
SS1G_02434	mitochondrial 2-oxoglutarate malate carrier protein	-	-	-	-	45.3	44.7
SS1G_06806	3-oxoacyl-[acyl-carrier protein] reductase	-	-	-	-	3.7	5.3
SS1G_10890	acetyl-CoA acetyltransferases	-	-	-	-	3.0	3.4
SS1G_04606	arsenite efflux transporter	-	-	-	-	-	2.8
SS1G_01231	sulfate permease	-	-	-	-	-	5.7
SS1G_01111	cytosine permease	-	-	-	-	-	3.6
SS1G_10204	mitochondrial carrier protein	-	-	-	-	-	2.2
SS1G_06998	peroxisomal, mitochondrial carrier protein	-	-	-	-	-	2.0
SS1G_04019	mitochondrial carrier protein	-	-	-	-	-	2.5
SS1G_08663	solute carrier protein	-	-	-	-	-	2.5
SS1G_05281	fatty acid transporter involves in acyl-CoA synthetase)	-	-	-	-	-	4.3
SS1G_06664	nucleoside transporter	3.7	5.7	5.7	4.3	2.8	-
SS1G_06212	nucleoside transporter	3.2	-	-	-	-	-
SS1G_04537	nucleoside transporter	-	-	-	-	2.6	-
SS1G_09667	nucleoside transporter	-	-	-	-	-	2.3

^a^Annotation based on the presence of conserved PFAM domains and BLAST reports

^b^Fold change relative to 0 h post inoculation (hpi). (-) No significant change in expression

More information about the genes can be found in Additional file 3: Table S3

To date, sclerin is the only selective phytotoxin reported from *S. sclerotiorum*. It has phytotoxic effects and causes necrotic and chlorotic tissue formation in *B. napus*, *B. juncea*, and *Sinapis alba* which are susceptible to sclerotinia stem rot disease, but not on a resistant species *Erucastrum gallicum* [77]. The genes involved in the sclerin synthesis pathway have not been reported; however, the presence of various genes encoding enzymes involved in the synthesis of known secondary metabolites in *S. sclerotiorum* and the comparably high levels of expression of these genes during infection suggests that, similar to *B. cinerea*, *S. sclerotiorum* has the capacity to secrete several different types of secondary metabolites. The transcriptome information reported in the current study will be useful in characterizing these secondary metabolite biosynthetic pathways.

#### 3. Detoxification

Plant pathogens must contend with various host biochemical defense mechanisms during the infection process. This can be achieved through avoidance (e.g. intracellular growth of pathogens to avoid extracellular phytoalexins), resistance (e.g. mutations that alter sensitivity to antimicrobial compounds) or detoxification (e.g. modification or degradation of host phytoalexins). The energy-dependent efflux of toxic phytochemicals by membrane-associated transporters is a general detoxification mechanism that is common in pathogens with broad host ranges [84]. A total of 33 genes encoding ATP-binding cassette (ABC) transporters and 218 major facilitator superfamily (MFS) transporters have been identified in the *S. sclerotiorum* genome [38]. As a group, the ABC and MFS transporters exhibit a wide range of specificities (polysaccharides, drugs, sugars, heavy metals, peptides, amino acids and inorganic ions); however, some have been implicated in the secretion of fungal toxins or the efflux of host phytoalexins [85].

In total, genes encoding 91 MFS transporters and 14 ABC transporters were up-regulated in the current study (Table 4). The contribution of ABC transporters to the ability of pathogens to tolerate phytoalexins is well known. In *B. cin*erea, the gene encoding the ABC transporter BcAtrB was induced during the early stages of infection and in the presence of camalexin. Inactivation of the *BcAtrB* gene lead to increased sensitivity to camalexin and reduced virulence [86]. BcAtrB has also been implicated in the transport of the phytoalexin resveratrol, certain antibiotics and fungicides, as well as the plant phenylpropanoid eugenol [87, 88]. The *S. sclerotiorum* orthologue of *BcAtrB* (SS1G_13659) was also up-regulated at 24-48 hpi in the current study and is likely to play a similar role in phytoalexin avoidance. While BcAtrA was not associated with *B. cinerea* virulence on bean, it is believed to be a multidrug transporter based on its ability to reduce the sensitivity of yeast to cycloheximide and catechol [89]. The *S. sclerotiorum* orthologue of *BcAtrA* (SS1G_06715) was slightly induced at 1 hpi. The ABC transporter AtrD has been implicated in resistance to demethylation inhibitor fungicides in *B. cinerea* [90] and *S. homeocarpa* [91], but the *S. sclerotiorum* orthologue (SS1G_02407) was not up-regulated during *B. napus* infection in the current study. The *S. sclerotiorum* orthologue of *BMR1* (SS1G_04483), which is involved in resistance to polyoxin and iprobenfos toxicants in *B. cinerea* [92], was up-regulated at 48 hpi in the current study.

*BcMFS1* encodes a MFS transporter in *B. cinerea* which is involved in detoxification of natural toxic compounds, such as camptothecin and cercosporin, and provides resistance to some fungicides, such as demethylation inhibitors (DMI) [93]. SS1G_12842 is orthologous to *BcMFS1* and was expressed, but not up-regulated, in the current study. Some of the MFS transporters up-regulated in the current study may be involved in detoxification, but this needs further investigation.

While transport of phytoalexins from the pathogen provides a mechanism to avoid the effects of host phytoalexins, enzymes may also be employed to permanently inactivate these compounds or transform them to a less toxic state. The gene encoding brassinin glucosyltransferase 1 (SsBGT1) (SS1G_09997) was up-regulated during infection of *B. napus* cultivar Surpass 400 leaves by *S. sclerotiorum* isolate UQ1280 [94]. *SsBGT1* was induced by plant phytoalexins, such as brassinin, and involved in detoxification of plant defense compounds via glucosylation [94]. This gene was also up-regulated at 24 and 48 hpi with expression levels 28 and 223-fold greater than the inoculum, respectively, in *S. sclerotiorum* 1980 in the current study.

The plant pathogen *Nectria haematococca* demethylates and detoxifies the pea phytoalexin, pisatin, by means of pisatin demethylase (PDA), a cytochrome P450 enzyme [95]. As such, PDA contributes to virulence of this fungus on pea [96]. SS1G_02340 encodes a cytochrome P450 enzyme (Table 3) with similarity to PDA from other plant pathogens, such as *Penicillium chrysogenum* and *Verticillium dahliae*, and was up-regulated at 1 to 12 hpi in the current study. It may be involved in the detoxification of structurally similar phytoalexins from *B. napus.*

Plant pathogens degrade aromatic compounds produced by plant defense systems, such as benzoic acid derivatives arising from the β-ketoadipate pathway [97]. The CYP53 family of cytochrome P450 enzymes play an essential role in this pathway through the hydroxylation of benzoic acid to 4-hydroxybenzoate. The first report of CYP53A1 enzyme function was from *Aspergillus niger* [98]. The enzyme encoded by the *Cochliobolus lunatus* orthologue of this gene, *CYP53A15*, was capable of para hydroxylation of benzoate [99]. The SS1G_01843 gene encodes a benzoate 4-hydroxylase and was up-regulated at 24 and 48 hpi in the current study and it may have a role in detoxification.

Propiconazole is a type of sterol DMI fungicide which inhibits the biosynthesis of ergosterol by targeting CYP51 (eburicol 14 alpha-demethylase) [100]. CYP51 is involved in the biosynthesis of fungal sterols which are required for membrane stability [101]. It has been speculated that overexpression of *CYP51* or its paralogues in *S. homoeocarpa* and *Monilinia fructicola* is one of the mechanisms that decrease sensitivity to DMI [91, 102]. SS1G_04805 is the paralogue of *S. homoeocarpa CYP51* and was up-regulated at 1 to 12 hpi in the current study. It may play a similar role in detoxifying phytoalexins.

Glucosinolates, found mainly in the Brassicaceae, and the more ubiquitous hydroxynitrile glycosides, are important plant defense compounds. These compounds undergo enzymatic transformation to release a wide variety of toxic metabolites upon tissue damage, including hydrogen cyanide, a potent inhibitor of cell respiration [103]. Cyanide hydratases were reported to have a role in detoxifying hydrogen cyanide in *B. cinerea* [104] and *Leptosphaeria maculans* [105]. In the current study, four genes encoded cyanide hydratases or cyanate hydrolases, SS1G_13754, SS1G_10174, SS1G_01652 and SS1G_11485 (Table 5), were significantly up-regulated during different infection stages from 1 to 48 hpi. These may be important for the detoxification of metabolites generated from glucosinolates during infection of *B. napus*.

**Table 5:** Description and expression of up-regulated genes involved in detoxification

Gene ID	Description^a^	Expression level (hpi)^b^					
		1	3	6	12	24	48
SS1G_01918	glutathione S-transferase	19.1	24.8	34.6	45.0	189.0	19.1
SS1G_10108	glutathione S-transferase	-	-	-	3.5	4.1	2.3
SS1G_08210	glutathione S-transferase	-	-	-	-	2.6	-
SS1G_04914	glutathione S-transferase	-	-	-	-	2.1	-
SS1G_07195	glutathione S-transferase	-	-	-	-	2.7	2.8
SS1G_14440	glutathione S-transferase	-	-	-	-	4.5	3.7
SS1G_09479	glutathione S-transferase	-	-	-	-	-	40.1
SS1G_08258	glutathione S-transferase with glutathionyl-hydroquinone reductase, ECM4 domain	-	-	-	-	3.1	2.4
SS1G_09997	UDP-glucuronosyl and udp-glucosyltransferase (brassinin)	-	-	-	-	28.0	223.2
SS1G_03517	UDP-glucoronosyl and udp-glucosyltransferase family protein	4.5	3.7	-	5.0	8.2	10.8
SS1G_13524	glucosyltransferase family	6.3	4.9	4.2	2.5	-	2.9
SS1G_13754	nitrilase-cyanide hydratase	3.2	3.5	3.1	2.2	4.6	4.4
SS1G_10174	nitrilase-cyanide hydratase	-	11.6	16.3	19.7	29.8	58.6
SS1G_01652	nitrilase-cyanide hydratase	-	-	-	-	3.4	2.3
SS1G_11485	cyanate lyase, cyanase superfamily	-	-	-	-	2.4	-
SS1G_10881	2-nitropropane dioxygenase	2.8	-	-	-	-	-
SS1G_14466	2-nitropropane dioxygenase	2.8	4.0	2.5	-	-	3.0
SS1G_00355	2-nitropropane dioxygenase	-	-	-	-	3.4	5.4
SS1G_11235	2-nitropropane dioxygenase	-	-	-	-	2.7	3.5

^a^Annotation based on the presence of conserved PFAM domains and BLAST reports

^b^Fold change relative to 0 h post inoculation (hpi). (-) No significant change in expression

More information about the genes can be found in Additional file 3: Table S3

Glutathione S-transferases (GST) are best known for their ability to conjoin the reduced form of glutathione to xenobiotic chemicals leading to their detoxification. Several genes encoding GSTs were significantly induced during different infection from 1 to 48 hpi with the majority being induced at 24 hpi (Table 5). SS1G_01918 was highly induced from 1 to 48 hpi with a peak expression level 189-fold greater than the inoculum. GSTs were first reported in plants because of their ability to detoxify herbicides [106]. Xenobiotic detoxification by this group of enzymes has also been reported in other fungi, such as *Phanerochaete chrysosporium* [107]. Bcgst1 from *B. cinerea* was the first GST reported in filamentous fungi; however, disruption of the *Bcgst1* gene indicated that the enzyme did not play a role in virulence [108]. Bcgst1 has been suggested to be a potential virulence factor as it is involved in tolerance against plant defense compounds, but the exact mechanism remains to be investigated. The orthologue of *Bcgst1* in *S. sclerotiorum* (SS1G_07195) was up-regulated at 24 and 48 hpi in the current study.

2-Nitropropane dioxygenase is an enzyme that catalyzes the oxidation of nitroalkanes, such as 2-nitropropane, into carbonyl compounds and nitrite. Nitroalkanes are toxic compounds [109] and 2-nitropropane dioxygenase is involved in detoxification of nitroalkanes in the *Trichoderma harzianum*-tomato interaction [110]. 2-Nitropropane dioxygenase is also involved in xenobiotic degradation by *Pseudomonas jeseenii* [111]. In the current study, four genes encoded 2-nitropropane dioxygenases, SS1G_10881, SS1G_14466, SS1G_00355 and SS1G_11235 (Table 5), were significantly up-regulated at different infection times from 1 to 48 hpi. These may be important for the detoxification of toxic nitroalkanes during infection of *B. napus*.

#### 4. Oxalic acid production

Oxalic acid (OA) is crucial for *S. sclerotiorum* infection and is required for suppression of host defenses [15], regulation of hydrolytic enzyme synthesis and activity [112], and the induction of processes leading to host colonization [113] and tissue necrosis [16, 27]. OA can be synthesized from a variety of precursors, but in fungi the most common mechanism is through the hydrolysis of oxaloacetate to form OA and carbon dioxide. An oxaloacetate acetyl hydrolase (OAH) has been characterized in *S. sclerotiorum* [114] and disruption of the *OAH* gene in *Aspergillus niger*, *B. cinerea* [115] and *S. sclerotiorum* leads to loss of oxalic acid production and reduced virulence [116]. In the current study, the *S. sclerotiorum OAH* gene (SS1G_08218) was expressed from 1 hpi, but only up-regulated at 48 hpi with 5- fold greater levels than the inoculum. *OAH* expression was also detected in the *S. sclerotiorum*– *P. vulgaris* system, but at 6 and 72 hpi, with relatively higher expression levels at the later time [40]. Interestingly, the gene encoding oxalate decarboxylase (SS1G_10796), an enzyme that degrades OA [117], was also induced at 24 and 48 hpi at 10 and 7-fold greater than the inoculum, respectively. Simultaneous expression of these genes with contrasting roles fits well with the biological functions of OA which are dependent on a balance between the biosynthesis and decomposition of OA to tightly regulate OA levels through the course of infection [27]. The results of the current study showing concurrent expression of *OAH* and the gene encoding oxalate decarboxylase are in agreement with Amselem et al. [38]. The acidic environment produced by OA has a significant role in virulence/pathogenesis of *S. sclerotiorum*. Release of OA causes a reduction in ambient pH, which stimulates hydrolytic enzyme production throughout the course of the infection [112, 118], as well as sclerotogenesis during its final stages [7, 119], though OA may not be the sole determinant affecting tissue acidification [120]. Interestingly, fine-tuning of OA levels through the activity of OA biogenic [121] and degradative [116] enzymes appears to be critical for early host-pathogen interactions as well, including compound appressorium formation and lesion expansion.

#### 5. Generation of reactive oxygen species

Reactive oxygen species (ROS), including superoxide, hydrogen peroxide (H_2_O_2_) and hydroxyl radicals, are produced by all aerobic organisms [122]. Plants generate ROS as part of the defense response against pathogen attack [123], but they may also be involved in cell proliferation and differentiation, signal transduction and ion transport [124]. Nicotinamide adenine dinucleotide phosphate (NADPH) oxidases (NOX) are key enzymes in oxidative burst activation resulting in ROS production [8, 125]. NOXs produce superoxide, an important precursor of several ROS, which is then converted to H_2_O_2_ by superoxide dismutase [125].

In filamentous fungi, NOX enzymes are involved in various aspects of differentiation, such as sexual reproduction and the formation of penetration structures [126]. In *B. cinerea* both BcNOXA and BcNOXB enzymes are involved in the formation of sclerotia and pathogenicity, while BcNOXB has been specifically implicated in events leading to penetration and BcNOXA in the spreading of lesions [125]. Importantly, NOX enzymes in *B. cinerea* do not play a role in ROS production [125]. Two genes encoding *S. sclerotiorum* NADPH oxidases, *SsNOX1* (SS1G_05661) and *SsNOX2* (SS1G_11172), have been reported [8]. *SsNOX1* is important for both virulence and fungal development and is also connected to oxalate production [8]. *SsNOX1* and *SsNOX2* were expressed, but not up-regulated in the current study. Interestingly, Kim et al. [8] proposed that the bicupin domain enzyme encoded by SS1G_10796 may be an oxalate oxidase. Oxalate oxidase catalyzes the generation of hydrogen peroxide (H_2_O_2_) from oxalate [8] suggesting that ROS generated by *S. sclerotiorum* during pathogenesis may be oxalate-mediated. SS1G_10796 was up-regulated at 24 and 48 hpi in the current study which coincided with the beginning of necrotic stage. This is in agreement with the previous study that oxalate-induced H_2_O_2_ in the host has a role in programmed cell death [16].

#### 6. Signaling

##### 6.1 Transcription factors

Transcription factors (TFs) are essential players in the regulatory networks that govern developmental processes and the deployment of pathogenicity factors during infection. In the current study, many genes encoding diverse putative TFs were up-regulated at different stages of the infection from 1 to 48 hpi (Table 6). Several of these encoded zinc-binding TFs from the major families prevalent in fungi, namely, Cys2 His2, Cys4, and Zn2 Cys6 families [127].

**Table 6:** Description and expression of up-regulated genes encoding transcription factors

Gene ID	Description^a^	Expression level (hpi)^b^					
		1	3	6	12	24	48
*1. Zn2Cys6 (C6)*							
SS1G_06255	Zn2 Cys6 transcription factor	7.0	4.6	3.9	2.4	-	-
SS1G_14383	Zn2 Cys6 transcription factor	3.5	4.4	3.5	2.1	-	-
SS1G_08819	Zn2 Cys6 transcription factor	3.6	2.5	2.1	3.0	-	-
SS1G_06907	Zn2 Cys6 transcription factor	4.1	4.1	3.3	-	-	-
SS1G_02793	Zn2 Cys6 transcription factor	6.0	3.0	-	-	-	-
SS1G_10324	Zn2 Cys6 transcription factor	2.0	-	-	-	2.0	-
SS1G_02851	Zn2 Cys6 transcription factor	2.2	-	-	-	-	-
SS1G_12532	Zn2 Cys6 transcription factor	2.2	-	-	-	-	-
SS1G_12799	Zn2 Cys6 transcription factor	-	2.5	2.5	-	-	-
SS1G_11949	Zn2 Cys6 transcription factor	-	2.7	2.5	-	-	-
SS1G_00732	Zn2 Cys6 transcription factor	-	2.4	-	5.0	-	-
SS1G_00398	Zn2 Cys6 transcription factor	-	4.1	-	-	-	4.8
SS1G_01905	Zn2 Cys6 transcription factor	-	-	-	-	2.7	-
SS1G_06876	Zn2 Cys6 transcription factor	-	-	-	-	2.3	-
SS1G_00170	Zn2 Cys6 transcription factor	-	-	-	-	2.3	-
SS1G_02339	Zn2 Cys6 transcription factor	-	-	-	-	2.0	-
SS1G_07143	Zn2 Cys6 transcription factor	-	-	-	-	2.7	-
SS1G_02791	Zn2 Cys6 transcription factor	-	-	-	-	2.9	3.0
SS1G_00392	Zn2 Cys6 transcription factor	-	-	-	-	2.2	2.2
SS1G_13144	Zn2 Cys6 transcription factor	-	-	-	-	2.1	3.0
SS1G_02054	Zn2 Cys6 transcription factor	-	-	-	-	6.6	36.9
SS1G_05109	Zn2 Cys6 transcription factor	-	-	-	-	6.7	7.2
SS1G_05755	Zn2 Cys6 transcription factor	-	-	-	-	2.6	3.8
SS1G_07003	Zn2 Cys6 transcription factor	-	-	-	-	-	4.6
SS1G_10447	Zn2 Cys6 transcription factor	-	-	-	-	-	2.5
SS1G_01353	Zn2 Cys6 transcription factor	-	-	-	-	-	4.1
SS1G_11367	Zn2 Cys6 transcription factor	-	-	-	-	-	3.3
SS1G_08351	Zn2 Cys6 transcription factor	-	-	-	-	-	2.1
SS1G_03775	Zn2 Cys6 transcription factor	-	-	-	-	-	6.8
SS1G_00787	Zn2 Cys6 transcription factor	-	-	-	-	-	2.8
SS1G_01733	Zn2 Cys6 transcription factor	-	-	-	-	-	2.6
SS1G_04056	Zn2 Cys6 transcription factor	-	-	-	-	-	2.7
SS1G_11081	Zn2 Cys6 transcription factor	-	-	-	-	-	2.8
SS1G_09741	fungal specific transcription factor domain	2.2	3.7	3.9	2.7	-	-
SS1G_04333	fungal specific transcription factor domain	2.0	2.3	2.0	-	-	-
SS1G_12561	fungal specific transcription factor domain	2.3	2.1	-	-	-	-
SS1G_09823	fungal specific transcription factor domain	-	-	2.1	-	2.2	2.4
SS1G_04846	fungal specific transcription factor domain	-	-	-	-	2.6	4.0
SS1G_13882	fungal specific transcription factor domain	-	-	-	-	2.0	-
SS1G_13729	fungal specific transcription factor domain	-	-	-	-	2.4	-
SS1G_02758	fungal specific transcription factor domain	-	-	-	-	2.5	-
SS1G_08406	fungal specific transcription factor domain	-	-	-	-	2.3	-
SS1G_05809	fungal specific transcription factor domain	-	-	-	-	3.9	-
SS1G_04057	fungal specific transcription factor domain	-	-	-	-	-	2.4
SS1G_11395	fungal specific transcription factor domain	-	-	-	-	-	2.3
SS1G_06361	fungal specific transcription factor domain	-	-	-	-	-	2.1
*2. Cys2His2*							
SS1G_01109	C2H2 transcription factor	19.8	26.1	26.6	15.6	-	-
SS1G_01684	C2H2 transcription factor	2.6	3.1	2.7	2.0	-	-
SS1G_04676	C2H2 transcription factor	2.0	-	-	-	-	7.3
SS1G_07355	C2H2 transcription factor (Pac1)	-	-	-	-	-	8.4
SS1G_10532	C2H2 transcription factor	-	-	-	-	-	20.5
SS1G_00104	C2H2 binding site	-	-	2.5	3.1	-	5.4
SS1G_09588	C2H2 binding site	-	-	-	-	2.9	4.4
SS1G_06370	C2H2 binding site	-	-	-	-	-	2.1
SS1G_09499	C2H2 binding site	-	-	-	-	-	3.1
SS1G_07425	C2H2 binding site	-	-	-	-	-	4.3
SS1G_06044	C2H2 binding site	-	-	-	-	-	2.3
*3. basic leucine zipper (bZIP)*							
SS1G_10244	bZIP transciption factor	4.4	5.2	-	5.6	-	-
SS1G_01411	bZIP transciption factor	-	-	-	-	2.0	-
SS1G_00816	bZIP transciption factor	-	-	-	-	-	5.4
SS1G_06075	bZIP transciption factor	-	-	-	-	-	7.7
*4. Others*							
SS1G_14328	transcription regulator BDF1	2.7	6.5	5.5	3.6	-	-
SS1G_11030	CP2 transcription factor	4.9	3.2	2.5	-	-	-
SS1G_04107	MYB family transcription factor	2.5	2.9	-	-	-	-
SS1G_06257	pex2/pex12 superfamily, zinc finger of C3HC4-type	3.0	2.4	-	-	-	-
SS1G_07430	zinc finger domain	3.0	-	-	-	-	2.8
SS1G_03881	regulator of G protein signaling domain protein (RGS) (GTPase activating proteins (GAPs)	2.0	-	-	-	-	-
SS1G_14385	CBF/Mak21 transcription factor	2.4	-	-	-	-	-
SS1G_03280	helix-loop-helix dna-binding protein (HlH)	2.4	-	-	-	-	-
SS1G_10206	CHY and ring, zinc finger protein	-	2.1	-	-	-	-
SS1G_04050	NF-X1 zinc finger transcription factor	-	3.0	-	-	-	-
SS1G_11663	large tegument protein UL36 and similar to TFIIIC transcription initiation factor complex subunits Tfc3	-	-	2.1	2.0	-	-
SS1G_03992	transcription mediator subunit Med12	-	-	2.2	-	-	-
SS1G_13930	Bro1-Alix- like domain and pH-response regulator protein	-	-	-	-	2.3	-
SS1G_07542	RING-H2 zinc finger protein	-	-	-	-	2.3	-
SS1G_06124	mads-box mef2 type transcription factor (SRF type)	-	-	-	-	-	3.5
SS1G_03098	homeobox transcription factor	-	-	-	-	-	2.2
SS1G_03835	homeobox C2H2 transcription factor	-	-	-	-	-	3.1
SS1G_06987	yippee zinc-binding protein	-	-	-	-	-	2.4
SS1G_01859	MYB dna-binding containing domain protein	-	-	-	-	-	6.2
SS1G_08831	(vWA) transcription factor (Von Willebrand factor type A)	-	-	-	-	-	2.3
SS1G_09890	RAP transcription factor	-	-	-	-	-	2.6
SS1G_02756	transcription factor protein	-	-	-	-	-	8.5
SS1G_10304	tetratricopeptide (TPR) repeat transcriptional corepressor	-	-	-	-	-	2.3
SS1G_13511	lipopolysaccharide (LPS)-induced transcription factor and LITAF-like zinc ribbon domain	-	-	-	-	-	2.7

^a^Annotation based on the presence of conserved PFAM domains and BLAST reports

^b^Fold change relative to 0 h post inoculation (hpi). (-) No significant change in expression

More information about the genes can be found in Additional file 3: Table S3

The gene encoding the Pac1 zinc finger domain transcription factor (SS1G_07355) was up-regulated 8-fold at 48 hpi in the current study. *Pac1* is an orthologue of *Aspergillus nidulans PacC* and controls pH-sensitive gene expression. Its activity was required for the control of a variety of physiological and pathogenesis-related processes in *S. sclerotiorum* [7]. In response to increasing ambient pH, Pac1 triggers OA biosynthesis leading a reduction in pH; this in turn causes an increase in *SsPG1* expression and promotes sclerotial development [119]. By affecting ambient pH, Pac1 is thought to play a role in OA and PG accumulation and therefore its activity is critical for *S. sclerotiorum* pathogenesis.

Biosynthesis of the *B. cinerea* phytotoxin, botrydial, is regulated by the Cys2 His2 zinc finger TF *BcCRZ1* [128], as well as upstream signaling components phospholipase C (*BcPLC1*) and calcineurin [129]. Four genes encoding phospholipase C-like enzymes (SS1G_03548, SS1G_05073, SS1G_08747 and SS1G_13589) were up-regulated at 48 hpi in the current study, suggesting they might be involved in secondary metabolite biosynthesis signaling pathways or events related to the later stages of the infection.

The *Penicillium roqueforti Pcz1* gene encodes Zn2 Cys6 TF which contributes to the regulation of growth, conidiation, and conidial germination [130]. *BcYOH1* from *B. cinerea* encodes a Cys2 His2 TF which is involved in the regulation of secondary metabolite synthesis [131]. A number of genes encoding zinc cluster TFs similar to *BcYOH1* and *Pcz1* were differentially expressed at various stages of infection in the current study, including SS1G_10532, SS1G_01109 and SS1G_02054 (Table 6). Several genes encoding other types of TFs involved in fungal development were also up-regulated and are discussed below.

##### 6.2 Phosphorylation-dependent signaling

The *S. sclerotiorum* genome contains many different types of kinases which are involved in signaling pathways, including the G protein-coupled receptor, MAP kinase, heterotrimeric G protein, cAMP, and Ca^2+^-related signaling pathways [38]. A number of genes associated with these pathways were up-regulated during infection in the current study (Table 7) and are discussed below. While the induction of genes encoding signaling pathway components is not always necessary for activation of their associated pathways, it does imply that they may be involved in more critical aspects or regulatory checkpoints during the infection process.

**Table 7:** Description and expression of up-regulated genes involved in signaling or gene regulation

Gene ID	Description^a^	Expression level (hpi)^b^					
		1	3	6	12	24	48
*1. Protein Kinases*							
SS1G_10983	MAPKK kinase (SsBCK1)	4.7	5.4	4.4	2.4	-	-
SS1G_11525	protein kinase-like protein	2.5	3.6	6.9	6.1	-	-
SS1G_00606	MAPKK kinase (SsSTE11)	2.1	2.2	-	-	-	2.1
SS1G_08085	AGC protein kinase (SsRIM15)	-	2.1	2	-	-	-
SS1G_03455	HAL family protein kinase	-	-	-	-	3.3	3.7
SS1G_06203	CAMK protein kinase (SsRCK2)	-	-	-	-	-	2.3
SS1G_10021	CAMK protein kinase (SsCMK2)	-	-	-	-	-	4.1
SS1G_06542	ran1-like protein kinase (SsSKS1)	-	-	-	-	-	2.3
SS1G_09511	protein kinase (Ss-Other-03)	-	-	-	-	-	5.8
SS1G_14212	Funk1 serine threonine-protein kinase	2.1	-	-	-	-	-
SS1G_12423	Funk1 serine threonine-protein kinase	2.3	2.4	-	-	-	-
SS1G_09355	Funk1 serine threonine-protein kinase	2.1	2.3	-	-	-	-
SS1G_10091	two-component histidine protein kinase (SHK1)	2.6	-	-	-	-	-
*2. Phosphatases*							
SS1G_11340	tyrosine phosphatase	2.6	2.1	2.2	-	-	2
SS1G_01711	protein phosphatase type 1 complex subunit hex2 reg1	3.3	3.2	2.2	-	-	-
SS1G_04320	tyrosine-protein phosphatase non-receptor type partial	2.1	3.5	2.5	-	-	-
SS1G_06382	protein phosphatase regulator	2.1	-	-	-	-	-
SS1G_12383	histidine acid phosphatase	-	2.8	-	-	-	-
SS1G_08513	serine threonine-protein phosphatase PP2A	-	-	-	-	-	2.8
SS1G_10466	diketo-5-methylthio-1-phosphopentane phosphatase	-	-	-	-	-	2.5
*3. GTPase/GTP-binding*							
SS1G_03234	rho GTPase activator	3.1	5	4.6	4.8	-	-
SS1G_06571	GTP-binding protein rho2	3.2	3	2.1	-	-	-
SS1G_01564	nuclear GTP-binding protein NUG1	2.9	-	-	-	-	-
SS1G_04075	ARF GTPase activator	-	-	-	-	-	2.5
SS1G_10333	GTP-binding protein	-	-	-	-	-	2.3
SS1G_08371	CLP1 GTPase	-	-	-	-	-	2.9
SS1G_13589	phosphatidyl inositol phospholipase C (PL-PLC)	-	-	-	-	-	2.1
SS1G_03548	phosphatidyl inositol phospholipase C (PL-PLC)	-	-	-	-	-	2.1
SS1G_05073	phosphatidyl inositol phospholipase C (PL-PLC)	-	-	-	-	-	5
SS1G_08747	phosphatidyl inositol phospholipase C (PL-PLC)	-	-	-	-	-	9
*4. Other*							
SS1G_06667	sir2 chromatin regulatory protein	2.1	3	2.6	2.1	-	-
SS1G_06180	Pal1 morphogenesis-related protein	9.5	8.7	7.9	4.5	-	-
SS1G_04402	Arrestin (chitin synthesis regulation)	-	-	-	-	2.1	-
SS1G_03525	ankaryin repeat protein	-	-	-	-	2.7	-
SS1G_04325	SUR7/Pal1 family (pH-response regulator)	-	-	-	-	-	2.2
SS1G_10456	SUR7/Pal1 family (pH-response regulator)	-	-	-	-	-	12.6
SS1G_09665	inositol-pentakisphosphate 2-kinase	-	-	-	-	-	2.8
SS1G_00378	SRP19 signal recognition particle protein	-	-	-	-	-	3
SS1G_08048	Sok1 cAMP-mediated signaling protein	-	-	-	-	-	2.7

^a^Annotation based on the presence of conserved PFAM domains and BLAST reports

^b^Fold change relative to 0 h post inoculation (hpi). (-) No significant change in expression

More information about the genes can be found in Additional file 3: Table S3

Two-component histidine kinases are known to be involved in regulating responses to environmental stimuli in fungi and bacteria [132]. The gene encoding the two-component sensor histidine protein kinase *Shk1* (SS1G_10091) was up-regulated only at the beginning of the infection (1 hpi) in the current study. *Shk1* was previously shown to have a role in hyphal growth and sclerotial formation in *S. sclerotiorum,* but was not required for pathogenicity on plant leaves [10].

The suite of genes encoding eukaryotic protein kinases in *S. sclerotiorum* has been catalogued [11]. Genes encoding two MAPKKK genes (SS1G_00606 and SS1G_10983) were up-regulated at 48 and 1-6 hpi, respectively. SS1G_00606 is an orthologue of *STE11* in *S. cerevisiae* and belongs to the *S. sclerotiorum STE11*-like MAPKKK family, while SS1G_10983 is an orthologue of *BCK1* in the yeast cell wall integrity pathway. Mutation of the *STE7* and *STE11* orthologues in *B. cinerea* [133] or *MST7* and *MST11* in *M. grisea* [134] disrupted the formation of infection structures leading to loss of pathogenicity. SS1G_10021 belongs to the CAMK1 family in *S. sclerotiorum* and is orthologous to the *S. cerevisiae* CMK1/CMK2 kinases. SS1G_10021 was up-regulated at 48 hpi in the current study. CAMKs were reported to have a role in the regulation of cell wall integrity and the response to oxidative stress [135]. A gene (SS1G_03455) orthologous to *S. cerevisiae SAT4*/*HAL4* was induced at 24-48 hpi in this study. HAL family kinases play a role in the regulation of membrane permeases which are responsible for amino acid and glucose transport [136]. Genes encoding two other protein kinases, SS1G_09511 and SS1G_06542, were both significantly up-regulated at 48 hpi in the current study. The kinase encoded by SS1G_09511 has some similarity to PHO85 (SS1G_07226), a cyclin-dependent kinase involved in the regulation of cell division in response to environmental stresses [137]. SS1G_06542 is an ortholog of *SHA3*/*SKS1*, which is involved in integration of the response to glucose with hyphal development [138]. The FunK1 protein kinases are similar to eukaryotic protein kinases, but are only found in multicellular fungi [139]. The *S. sclerotiorum* genome contains three members of the FunK1 family (SS1G_09355, SS1G_12423 and SS1G_14212), all of which were up-regulated at the earliest stages of the infection.

SS1G_06571, SS1G_03234 and SS1G_10333 encode proteins annotated as having GTPase or GTP-binding activity and were first up-regulated at 1, 1 and 48 hpi, respectively. In *S. sclerotiorum*, the small GTPase Rap-1 is involved in mediating the inhibitory actions of cAMP on the SMK1 MAPK signaling cascade and events leading to sclerotial development [9]. GTP-binding proteins belonging to the Ras superfamily also play a role in MAPK inhibition as effectors acting downstream of cAMP [9]. Other studies have shown that in addition to Ras, other small GTPases, such as Rap-1 and Rho/Rac/Cdc42, also have important roles in transmitting signals via activation of MAPK cascades [140].

Protein dephosphorylation is also employed to both activate and attenuate kinase-dependent signaling pathways. Calcineurin, a Type 2B serine/threonine phosphatase is required for proper sclerotial formation and hyphal cell wall formation [12]. The Type 2A serine/threonine phosphatase (PP2A) encoded by SS1G_08489 (PPH1) was shown to play a role in several aspects of *S. sclerotiorum* pathogenesis including hyphal growth, infection cushion formation, sclerotia development and synthesis of secondary metabolites such as melanin [13]. The genes encoding calcineurin and PPH1 were not up-regulated in the current study; however, a possible PPH1 paralogue (SS1G_08513) which encodes a serine/threonine phosphatase with a PP2A catalytic subunit was up-regulated at 48 hpi. It is possible that SS1G_08513 complements the function of PPH1in the *S. sclerotiorum* infection process, but characterization of its precise roles awaits further study. The SMK1 MAPK also positively regulates PPH1 activity through a nitrous oxide-dependent mechanism [13].

#### 7. Development

As is the case in most multi-cellular pathogens, *S. sclerotiorum* undergoes dramatic morphological and biochemical changes as it passes through the various stages of the infection process. The *S. sclerotiorum* genome contains orthologues of *M. oryzae* genes that are involved in infection structure production and penetration [38]. Among them, SS1G_13339, SS1G_10311 and SS1G_11468 were up-regulated in the current study (Table 8). SS1G_10311 and SS1G_11468 are orthologous to *mas2* and *mas3* in *M. oryzae*, respectively, where they play a role in appressoria formation during the very early stages of infection [141]. However, in the current study SS1G_10311 was up-regulated at 3 and 12 hpi and SS1G_11468 was up-regulated at 6-48 hpi, while SS1G_13339 was induced only at 48 hpi, suggesting that they may have alternate roles in this necrotrophic pathogen.

**Table 8:** Description and expression of up-regulated genes involved in fungal development

Gene ID	Description^a^	Expression level (hpi)^b^					
		1	3	6	12	24	48
*1. Reserve Mobilization*							
SS1G_05192	acid trehalase	-	-	-	-	6.7	5.8
SS1G_01494	1,3-alpha-glucanase/mutanase	-	-	-	-	-	30.7
SS1G_09861	1,3-alpha-glucanase/mutanase	-	-	-	-	-	15
*2. Cell Wall*							
*2.1 Turnover*							
SS1G_05454	chitinase	-	-	2.4	-	3.5	2.7
SS1G_11700	chitinase	-	-	-	-	3.7	69.1
SS1G_05897	chitinase	-	-	-	-	3	2.6
SS1G_11304	chitinase	-	-	-	-	-	4.8
SS1G_08695	class III chitinase	-	-	-	5.8	8.2	11.7
SS1G_11212	class III chitinase	-	-	-	-	-	9.3
SS1G_12510	class V chitinase	-	-	-	-	11.2	66.9
SS1G_09403	alpha-N-acetylglucosaminidase	-	-	-	-	2.9	-
SS1G_12837	beta-N-acetylglucosaminidase	-	-	-	-	2.3	-
SS1G_10038	beta-N-acetylglucosaminidase	-	-	-	-	-	3
SS1G_04898	polysaccharide (chitin) deacetylase	-	-	15.2	-	-	12.7
SS1G_01131	polysaccharide (chitin) deacetylase	-	-	-	-	3	3.6
SS1G_00642	polysaccharide (chitin) deacetylase	-	-	-	-	-	8.3
SS1G_12836	N-acetylglucosamine-6-phosphate deacetylase	-	-	-	-	-	6.7
SS1G_01229	exo-beta 1,3 glucanase	-	-	-	-	3.4	22.8
SS1G_09858	exo-beta 1,3 glucanase	-	-	-	-	-	2.8
SS1G_12930	GPI-anchored cell wall beta-1,3-endoglucanase	8.5	6.4	3.9	-	-	2.2
SS1G_04852	GPI-anchored cell wall beta-endoglucanase	2.1	-	-	-	-	-
*2.2 Biosynthesis*							
SS1G_04969	glycosyl transferase (cell wall synthesis)	2.2	-	-	-	-	-
SS1G_04062	glycosyl transferase (cell wall synthesis)	-	3.6	3.8	-	-	-
SS1G_07313	lipopolysaccharide biosynthesis protein	-	-	-	-	-	2.4
*3. Other*							
SS1G_02742	heterokaryon incompatibility protein	3.8	3.6	3.1	2.8	-	-
SS1G_02744	heterokaryon incompatibility protein (SEC1)	3.7	5.5	-	5	3	3
SS1G_02602	heterokaryon incompatibility protein	2.3	-	-	-	-	-
SS1G_03889	heterokaryon incompatibility protein	-	3.2	-	-	-	-
SS1G_11165	heterokaryon incompatibility protein	-	3.3	2.2	-	-	-
SS1G_06800	heterokaryon incompatibility protein	-	3.6	-	-	-	-
SS1G_08974	heterokaryon incompatibility protein (WD40 repeat)	-	-	-	-	2.7	-
SS1G_06855	heterokaryon incompatibility protein	-	-	-	-	5.2	2.4
SS1G_12973	heterokaryon incompatibility protein	-	-	-	-	-	2
SS1G_09167	heterokaryon incompatibility protein	-	-	-	-	-	2.5
SS1G_11315	heterokaryon incompatibility protein	-	-	-	-	-	4
SS1G_07526	ferritin-like sexual development protein	3	2.5	2.5	-	-	23.9
SS1G_04316	acyltransferase (hard surface induced)	2.5	-	-	-	-	-
SS1G_10311	DUF cell surface protein (MAS2 orthologue)	-	2.1	-	2.5	-	-
SS1G_11468	CAS1 appressorium specific protein (MAS3 orthologue)	-	-	2.9	7.3	12.9	10.5
SS1G_14127	gamma-glutamyltranspeptidase (SsGGT1)	-	-	3.4	6.5	4.8	2.7
SS1G_05330	gamma-glutamyltranspeptidase	-	-	-	-	2.1	-
SS1G_12877	conidiation-specific expression protein	-	-	-	-	2.3	2.3
SS1G_12133	predicted protein (SSP2)	-	-	-	-	4.7	9.6
SS1G_07404	predicted protein (Ss-Rh1)	-	-	-	-	4.9	3.8
SS1G_01614	G protein-coupled receptor (SOP1)	-	-	-	-	17.7	17.1
SS1G_07626	Velvet family	-	-	-	-	-	5.2
SS1G_13339	choline carnitine O-acyltransferase	-	-	-	-	-	3.4
SS1G_02422	UDP-galactopyranose mutase (GLF)	-	-	-	-	-	3.9
SS1G_10940	gamma-glutamyltranspeptidase	-	-	-	-	-	4
SS1G_14065	predicted protein (SSP1)	-	-	-	-	-	47

^a^Annotation based on the presence of conserved PFAM domains and BLAST reports

^b^Fold change relative to 0 h post inoculation (hpi). (-) No significant change in expression

More information about the genes can be found in Additional file 3: Table S3

Morphological changes can also be triggered by environmental cues. The enzyme γ-glutamyl transpeptidase regulates glutathione levels and in turn cellular redox potential. In *S. sclerotiorum*, the γ-glutamyl transpeptidase encoded by SS1G_14127 (SsGgt1) plays a role in the production of compound appressoria during host penetration as well as in the development of sclerotia, but is not necessary for host colonization and symptom development [24]. In the current study, SS1G_14127 was up-regulated from 6-48 hpi, while genes encoding two other γ-glutamyl transpeptidases (SS1G_05530 and SS1G_10940) were up-regulated during the later stages at 24 and 48 hpi, respectively.

The *SSP1* gene (SS1G_14065) was up-regulated 47-fold at 48 hpi in the current study. The *SSP2* gene (SS1G_12133), a paralogue of *SSP1*, was also up-regulated in the later stages of the infection. SSP1 is a sclerotium-specific protein that is associated with sclerotial and apothecial development and is only detected during sclerotial formation [142]. The up-regulation of *SSP1* and *SSP2* at 24-48 hpi is an indication of the onset of sclerotia development at these time points in the current study. *SOP1* is similar to microbial opsins, a component of the photosensory system, and is also required for sclerotial development, as well as growth and virulence in *S. sclerotiorum. SOP1* was first induced at early stages of infection in *A. thaliana* and then more so at the sclerotial development stage (3 days post inoculation) [143]. In the current study, the gene encoding SOP1 (SS1G_01614) was highly up-regulated (17-fold) at 24 and 48 hpi suggesting that this protein also contributes to the sclerotial development program in the *S. sclerotiorum* - *B. napus* pathosystem. Members of the velvet protein family coordinate fungal differentiation processes, including the formation of spores, sclerotia and fruiting bodies [144]. SS1G_07626 encodes a velvet protein and was induced only at 48 hpi indicating that it may also be involved in sclerotogenesis in *S. sclerotiorum.* Another gene, SS1G_07404 (*Ss-Rhs1*, *Sclerotinia sclerotiorum* rearrangement hotspot repeat 1), was up-regulated at 24 and 48 hpi in the current study and has been reported to be involved in sclerotial development and important for virulence in *S. sclerotiorum* [145].

MADS-box proteins are a conserved family of TFs and are involved in the regulation of a wide variety of functions including primary metabolism, cell cycle and cell identity [146]. A gene encoding a MADS-box TF (SS1G_06124) was reported as being a component of the mating process in *S. sclerotiorum* [38] and was up-regulated 3-fold at 48 hpi in the current study. The MADS-box TF SsMADS (SS1G_05588) is required for growth and virulence [146]. Homeobox genes regulate aspects of anatomical development and in fungi are involved in hyphal growth, appressorium formation or conidia production [147]. In the current study, a gene (SS1G_03835) encoding a homeobox C2H2 TF was expressed at 48 hpi, but the precise function of this gene in *S. sclerotiorum* needs to be characterized. A homeobox TF (BcHOX8) that plays a role in the vegetative growth and conidiogenesis has been reported in *B. cinerea* [148].

In fungi, programmed cell death associated with vegetative incompatibility is determined by the interactions of proteins containing heterokaryon incompatibility (HET) domains [149]. In the current study, 11 genes encoding heterokaryon incompatibility proteins were up-regulated at some point during the infection with one group induced during the early stages (SS1G_02742, SS1G_02602, SS1G_03889, SS1G_11165 and SS1G_06855) and a second group that was induced during the later stages (SS1G_08974, SS1G_06855, SS1G_12973, SS1G_09167 and SS1G_11315) (Table 8). Some HET domain-containing protein coding genes in *S. sclerotiorum* are paralogues of HET-E-1 family genes of *Podospora anserina* [38, 150], but these were not up-regulated in the current study. The role of HET domain-containing proteins in *S. sclerotiorum* and *B. cinerea* speciation has also been suggested [38]. Some *S. sclerotiorum* orthologues of *A. nidulans* genes involved in mating and fruiting body development, such as SS1G_09861 and SS1G_07526 [38], were also up-regulated in the current study. Additionally, SS1G_06124 (transcription factor) and SS1G_00606 (STE 11 kinase), which are also orthologues of *A. nidulans* genes involved in mating process signaling pathways [38], were significantly induced in the current study.

Mobilization of storage reserves, including those derived from cell wall turnover and reconstruction, accompanies major morphological phase transitions, such as sclerotial formation. Trehalose is a common storage carbohydrate in fungi and a gene encoding an acid trehalase (SS1G_05192) was induced at the later stages of the infection. Alpha-1,3-glucan (mutan) is a component of the fungal cell wall, but is also considered to be a major energy reserve [151]. Two genes encoding 1,3-alpha-glucanase/mutanase (SS1G_01494 and SS1G_09861) were sharply induced (15 and 30 fold) at 48 hpi. The expression of these genes closely coincided with the expression of a wide variety of genes encoding enzymes capable of degrading fungal cell walls, including chitinases, chitin deacetylases, N-acetylglucosaminidases and various endo-glucanases. In another study, 19% of all *S. sclerotiorum* genes encoding fungal cell wall degrading enzymes were up-regulated during infection [49]. They hypothesized that these enzymes were involved in cell wall reorganization or rearrangement as the pathogen progressed through different developmental stages.

#### 8. Secreted effectors

*S. sclerotiorum* secretes a large repertoire of various effector proteins that may be involved in aspects of pathogenesis or virulence [44]. Several of these, and others, were found to be up-regulated during infection of *B. napus* in the current study (Table 9) and are discussed below.

**Table 9:** Description and expression of up-regulated genes encoding putative effector proteins

Gene ID	Description^a^	Expression level (hpi)^b^					
		1	3	6	12	24	48
SS1G_08557	salicylate hydroxylase	4.1	5.4	5.1	3.6	5.1	10.8
SS1G_00849	22kda glycoprotein (AltA-1 allergen)	7.4	3.2	-	-	-	7.1
SS1G_10096	cerato-platanin (PF07249)	3.8	3.7	-	-	-	3.3
SS1G_07295	cfem domain-containing protein	2	-	-	-	3.9	4.8
SS1G_12336	chitin binding protein	-	3.3	-	-	-	-
SS1G_12509	LysM domain protein	-	-	-	3.8	11.8	35.4
SS1G_03611	cysteine-rich protein	-	-	-	-	85.1	247
SS1G_11912	npp1 domain protein (NEP2)	-	-	-	-	5.8	8.2
SS1G_03282	serine protease inhibitor	-	-	-	-	-	2.2
SS1G_02904	cyanoVirin-N homology (SsCVNH)	-	-	-	-	-	3.9
SS1G_00263	protein unique to *S. sclerotiorum* and *B. cinerea* (Ssv263)	-	-	-	-	-	49.2
SS1G_02068	predicted protein (SsSSVP1)	-	-	-	-	-	21.5

^a^Annotation based on the presence of conserved PFAM domains and BLAST reports

^b^Fold change relative to 0 h post inoculation (hpi). (-) No significant change in expression

More information about the genes can be found in Additional file 3: Table S3

One of the hallmarks associated with *S. sclertoriorum* infection is the rapid onset of necrosis. Two *S. sclerotiorum* necrosis and ethylene-inducing protein (NEP) proteins (SsNEP1 and SsNEP2) were characterized by Bashi et al. [30] and their necrosis-inducing activity demonstrated. In that study, both genes were induced at the mid to later times in the infection with *SsNEP2* being expressed at much higher levels than *SsNEP1*. This is in agreement with the current study, the *SsNEP2* gene (SS1G_11912) was induced at 24 and 48 hpi. Orthologues of these genes are also present in *B. cinerea* (*BcNEP1* and *BcNEP2*) and both proteins are capable of inducing necrosis in the host plants [152]. Cerato-plantanins are small, hydrophobic, secreted proteins found in many fungal phyto-pathogens and have been shown to induce plant defenses leading to systemic acquired resistance [153]. In *B. cinerea*, cerato-platanin is one of the most abundant secreted proteins and elicits a strong hypersensitive response in the host plant leading to localized necrotic lesions [154]. An *S. sclerotiorum* gene encoding cerato-platanin (SS1G_10096) was up-regulated at both the early and later stages of the infection in the current study. SsSSVP1 (SS1G_02068) encodes a small secreted, cysteine-rich protein that induces plant cell death by interfering with host energy metabolism and, as such, plays an important role in virulence in *S. sclerotiorum* [155]. In the current study, SS1G_02068 was up-regulated 21-fold at 48 hpi. In the *S. sclerotiorum- A. thaliana* interaction, *SsSSVP1* showed significant up-regulation starting from 3 hpi and slowly increased from 6 to 12 hpi [155], suggesting that the expression pattern of this gene could be host-dependent. As noted above, several hydrolytic enzymes, including certain polygalacturonases [54, 55] and xylanses [57], are also potent inducers of host necrosis.

A gene encoding a cysteine-rich protein with a CFEM (common fungal extracellular membrane) domain (SS1G_07295) did not show significant induction in a previous study conducted on a number of host plants [44], whereas in the current study it was induced between 2 and 4.8 fold throughout the course of the infection supporting the notion that expression of effector genes in *S. sclerotiorum* may be host-dependent. In *Magnaporthe grisea*, the CFEM protein Pth11 is involved in appressorium development [156], while in *Candida* species CFEM proteins were involved in biofilm formation and iron acquisition [157]. *SsCVNH* (SS1G_02904), which encodes a small, cysteine-rich, secreted protein with a CyanoVirin-N Homology (CVNH) domain, was previously predicted to be a candidate effector of *S. sclerotiorum* [44], and was shown to be important for infection, sclerotial development and growth of *S. sclerotiorum* [49]. *SsCVNH* was up-regulated at 48 hpi in the current study. Similarly, Lyu et al. [49] showed that *SsCVNH* was significantly up-regulated during the initial stages of sclerotial development occurring at 3 days post-inoculation. The induction of *SsCVNH* coincided with that of *Pac1* which is also involved in sclerotial development through OA-mediated pH reduction, suggesting that the expression of *SsCVNH* might also be pH-dependent. A gene encoding another cysteine-rich protein (SS1G_03611) was one of the most highly up-regulated genes detected in the current study and exhibited a 247-fold increase in expression at 48 hpi, while a gene encoding a protein unique to *S. sclerotiorum* and *B. cinerea* (SS1G_00263) was induced 49-fold at this time. The protein encoded by SS1G_00849 had none of the domains associated with fungal effectors; however, it is an orthologue of *Colletotrichum hingginsianum* effector candidate 91 (*CHEC91*) [158] and analogous to the *Alternaria alternate* AltA-1 allergen [44]. SS1G_00849 was significantly induced at 1–3 and 48 hpi in the current study supporting the view that it may also be a *S. sclerotiorum* effector.

Some effectors facilitate infection by abrogating the ability of the host to deploy appropriate defense responses. Lysin motif (LysM) effectors interfere with host detection of the pathogen by binding to and masking fungal cell wall–derived chitin fragments that would normally induce host defense responses [159]. A gene encoding a LysM protein (SS1G_12509) was up-regulated during the mid to later stages of the infection, while a gene encoding another chitin-binding protein (SS1G_12336) was up-regulated at 3 hpi. Salicylic acid is a signaling molecule required for the induction of plant defenses in response to many biotic and abiotic stresses. Enzymes that degrade salicylic acid are released by some fungal endophytes to suppress the deployment of such defenses [160]. In the current study, a gene encoding salicylate hydroxylase (SS1G_08557) was induced very early in the infection and remained up-regulated throughout, although this gene does not have signal peptide and it might be secreted through an alternative endoplasmic reticulum/Golgi-independent protein secretion mechanism.

## Conclusions

*S. sclerotiorum* deploys a wide variety of factors and complex strategies to establish disease and complete the infection of the host plant. Soon after encountering the surface of a suitable host plant, the pathogen releases enzymes that begin to digest the cuticle. The induction of *SsCuta* and genes encoding related cutinases/lipases at the earliest stages of the infection supports their role in cuticle penetration. The enzymatic degradation of other plant surface polymers and polysaccharides is also required for successful penetration and is carried out by an armory of hydrolytic enzymes, which were induced from 1 to 12 hpi in this study. These enzymes are released from the base of infection cushions which apply pressure to assist cuticle penetration. Induction of orthologous genes involved in appressorium formation in other fungi, such as *mas2* and *mas3* [38, 141] and *SsGgt1* [24] during the early stages of infection in the current study supports their role in the production of penetration-associated structures.

During penetration and subsequent proliferation through the host, the fungus must sequentially breach various layers of plant barriers. To do so, it releases a cocktail of hydrolytic enzymes, detoxification systems and effector proteins. A plethora of genes encoding hydrolytic enzymes were induced concurrent with cell wall and host plant tissue disruption and are required to release nutrients to facilitate spread of the pathogen. Noxious compounds liberated as a form of plant defense or through the activities of the pathogen itself must also be detoxified. This study revealed that *S. sclerotiorum* induces the expression of genes encoding a wide variety of ABC and MFS transporters, cytochrome 450 enzymes, GSTs., etc, during the infection that may allow it to contend with various host plant defense mechanisms and toxins.

Recent studies have identified a brief biotrophic phase within the apoplastic space immediately after cuticle penetration [3]. During infection of *B. napus* in the current study, this biotrophic stage might occur between 12 and 24 hpi since biotrophy-related effector genes, such as those encoding the LysM domain protein and salicylate hydroxylase, were up-regulated during this period. These proteins may assist with suppression of host-pathogen recognition and defense systems. However, the genes encoding SSITL [26] and chorismate mutase (SsCM1) [27], which also help to suppress plant defense responses during the biotrophic phase, were not induced in the current study. This discrepancy might indicate that *S. sclerotiorum* is armed with alternate, unelucidated, strategies to suppress host defenses to establish a biotrophic phase in different host plants.

The appearance of necrotic lesions at 24 hpi indicated that initial penetration and the biotrophic phase had ended by this time. The onset of the necrotrophic stage requires that a different set of genes be expressed, including those encoding hydrolytic enzymes, enzymes involved in secondary metabolite synthesis or toxins to trigger host programmed cell death. Numerous genes encoding enzymes involved in the synthesis of polyketides and non-ribosomal peptides were up-regulated throughout the infection, but more so during the later stages. Previous studies also suggested that the induction of genes encoding NEP proteins coincides with the beginning of the necrotrophic phase [161]. The expression of *SsNEP2* in the current study was induced beginning at 24 hpi, confirming that *S. sclerotiorum* had switched to the necrotrophic stage around this time.

The acidic environment resulting from OA accumulation is a critical step during the necrotrophic phase of *S. sclerotiorum*. In the present study, the genes encoding Pac1 and subsequently OAH which are indirectly and directly involved in OA biosynthesis, respectively, were up-regulated at 48 hpi, supporting the notion that OA accumulates during the necrotrophic phase of infection. The acidic environment produced by OA stimulates hydrolytic enzyme production, specifically SsPG1 [7, 112, 118, 119]. Induction of *acp1,* whose expression is sensitive to pH, is also regulated by Pac1 [21]. Interestingly, the concurrent expression of *OAH* and the gene encoding oxalate decarboxylase suggests that a balance between biosynthesis and decomposition of OA is required for tight regulation of OA levels through the course of infection [38]. In addition to its role in regulating the transition to the necrotrophic phase, OA also plays a role in suppression of the oxidative burst in the host plant during the early stages of the infection [15] and may therefore contribute to the establishment of a biotrophic phase. However, *OAH* expression was not induced during the early stages of infection in the current study suggesting that an alternative pathway for OA biosynthesis independent of OAH exists or more likely that a basal amount of OA is sufficient for suppression of oxidative burst.

In addition to the well-known effectors that have been mentioned above, a number of genes encoding other *S. sclerotiorum* effectors, including SsBi1 [20], SsCaf1 [25], SCat1 [18], SsSodI [19] and SsPemG1 [29] were expressed, but not significantly induced in the current study. The discrepancy might be due to host and isolate-dependent differences in the expression of these genes or differences in experimental design.

In summary, the current study revealed a vast set of genes encoding various hydrolytic enzymes, enzymes involved in secondary methobolite biosynthesis, proteins associated with detoxification systems and effector proteins that collectively facilitate the infection of *B. napus* by *S. sclerotiorum.* The present investigation gives a global view of the gene expression of *S. sclerotiorum* as it infects *B. napus* and provides a baseline for further characterization of important genes involved in the *S. sclerotiorum*- *B. napus* and other host molecular interactions.

## Additional files

Additional file 1: Table S1. Oligonucleotides used for ddPCR. Sequences of the forward (F) and reverse (R) primers used for droplet digital PCR (ddPCR) to examine expression of select *Sclerotinia sclerotiorum* genes during infection of *Brassica napus*. (DOCX 17 kb)

Additional file 2: Table S2. Output summary generated by CLC Genomics Workbench of mapped Illumina reads against *S. sclerotiorum* isolate 1980 reference transcriptome. Account of the total and mapped Illumina reads generated from three biological replicates of libraries generated from *Sclerotinia sclerotiorum* mycelia at various times during infection of *Brassica napus*. (DOCX 22 kb)

Additional file 3: Table S3. List of up-regulated genes with BLAST2GO annotation. Description of all genes that were up-regulated in *Sclerotinia sclerotiorum* at various times during infection of *Brassica napus*. (XLSX 413 kb)

Additional file 4: Figure S1. Clustering of differentially expressed genes based on expression patterns relative to time of inoculation (time 0). Increasing intensity indicates greater fold change (red = positive; green = negative) for individual genes. The expression of *Sclerotinia sclerotiorum* genes at various times during infection of *Brassica napus* were used for cluster analysis and a heat map was generated. (PDF 208 kb)

## Abbreviations

ABC: ATP-binding cassette
CAZymes: Carbohydrate-active enzymes
CE: Carbohydrate esterase
CFEM: Common fungal extracellular membrane
CWDE: Cell wall degrading enzymes
ddPCR: droplet digital PCR
DMI: Demethylation inhibitor
EST: Expressed sequence tag
FDR: False discovery rate
GH: Glycoside hydrolase
GO: Gene ontology
GST: Glutathione S-transferase
MFS: Major facilitator superfamily
NADPH: Nicotinamide adenine dinucleotide phosphate
NRPS: Nonribosomal peptide synthase
OA: Oxalic acid
OAH: Oxaloacetate acetyl hydrolase
PCA: Principal component analysis
PCD: Programmed cell death
PG: Polygalacturonase
PKS: Polyketide synthase
ROS: Reactive oxygen species
RT: Reverse transcriptase
Ss1980: *Sclerotinia sclerotiorum* isolate 1980
TF: Transcription factor
TMM: Trimmed means of means
