# Supplementary material for: Changes in the Sclerotinia sclerotiorum transcriptome during infection of Brassica napus
Source: BMC Genomics. 2017 Mar 29;18:266. doi: 10.1186/s12864-017-3642-5 (PMC5372324; doi:10.1186/s12864-017-3642-5)
Supplement: Supplementary file 1 — Oligonucleotides used for ddPCR. Sequences of the forward (F) and reverse (R) primers used for droplet digital PCR (ddPCR) to examine expression of select Sclerotinia sclerotiorum genes during infection of Brassica napus. (DOCX 17 kb) [file 12864_2017_3642_MOESM1_ESM.docx]

Additional file 1: Table S1. Oligonucleotides used for ddPCR

**Gene ID Primer Sequences 5’>3’ Probe Sequence 5’>3’**

| SS1G_07027 | F: CAACTTGATCGACACACCTACT  R: GGATAACCATCGAGTGCGAATA | **FAM**/AGCTCCTCAAGGTTTGAATGTATCATGCA |
| --- | --- | --- |
| SS1G_07661 | F: CTCGCGGAACAACTGAGATT  R: GATAATCGACGCCGGTCATAG | **FAM**/AAGCCCTGCAATCTTCTCTCGGTT |
| SS1G_08104 | F: CCAATCCAAGCTAGTGCAGTTAAT  R: CCTTGGGCTCTGCAGGTA | **FAM**/AGCTGCAATCTTCATGGGTGATCCA |
| SS1G_08218 | F: CGCTCCAACAACTTGGTTATG  R: AGCCATCTCCTTTGAAGTGTAA | **FAM**/CTTGGTGCAGATGTTGGCTTGCTC |
| SS1G_10796 | F: AGGATGGGCAAGACAACAA  R: GCCAGTGTAACTCTCGGTATG | **FAM**/TGCGACTGAAATGGCTGGTGTAGA |
| SS1G_10167 | F: GGAAGCGTTCTCGATGGTAA  R: CTTCAAGGAGTGAGCGTAGAAG | **FAM**/ACGGTGGAAAGACCAAGCCAAAGT |
| SS1G_07355 | F: AGAAGTGCTGGTGGACTTAAC  R: GCGAGGTGTTGTTTCCTTTG | **FAM**/ACCGTTGGTTCTCCAACACCATCT |
| SS1G_14133 | F: CGGAGCATCAACCTACAAGAC  R: CTGGCTTTCCATCACCATCATA | **FAM**/CGGGAATGAGCAAGATGGTCAATGGA |
| SS1G_02486 | F: ATCCGGGTGCCTTCTTTAC  R: CAGTCTCATCTTCCATGCCATA | **FAM**/ATTTCGACAACAACGGCGTTTGGG |
| SS1G_05839 | F: CACACAGGAATCGGTATTGGA  R: CATCACCACCCACGGATTAG | **FAM**/CGCGCGCAATGTTTCAAAGTGGTT |
| SS1G_04652 | F: ACCGTCGTCGAGCCATATAA  R: TAGAGAGCCTCGTTGTCGATAC | **HEX**/TGGTCGAGAACTCTGACGAGACCT |

R indicates reverse primer and F forward primer
