# Supplementary material for: Changes in the Sclerotinia sclerotiorum transcriptome during infection of Brassica napus
Source: BMC Genomics. 2017 Mar 29;18:266. doi: 10.1186/s12864-017-3642-5 (PMC5372324; doi:10.1186/s12864-017-3642-5)
Supplement: Supplementary file 2 — Output summary generated by CLC Genomics Workbench of mapped Illumina reads against S. sclerotiorum isolate 1980 reference transcriptome. Account of the total and mapped Illumina reads generated from three biological replicates of libraries generated from Sclerotinia sclerotiorum mycelia at various times during infection of Brassica napus. (DOCX 22 kb) [file 12864_2017_3642_MOESM2_ESM.docx]

Additional file 2: Table S2. Output summary generated by CLC genomic workbench of mapped Illumina reads against *S. sclerotiorum* isolate 1980 reference transcriptome.

| Replicate | Sample | High-quality paired-end reads | Single reads mapped to reference | | % | | | Paired-end reads mapped to reference | % | |  |
| --- | --- | --- | --- | --- | --- | --- | --- | --- | --- | --- | --- |
| Rep 1 |  |  | |  | |  |  | | |  | |
|  | *S. sclerotiorum* | 2,816,990 | | 2,068,369 | | 73.42 | 1,807,130 | | | 64.15 | |
|  | 1h | 2,056,400 | | 1,225,180 | | 59.58 | 1,082,390 | | | 52.64 | |
|  | 3h | 900,572 | | 518,607 | | 57.59 | 455,912 | | | 50.62 | |
|  | 6h | 1,284,272 | | 709,409 | | 55.24 | 633,452 | | | 49.32 | |
|  | 12h | 747,264 | | 445,373 | | 59.60 | 398,704 | | | 53.36 | |
|  | 24h | 1,474,458 | | 892,355 | | 60.52 | 802,708 | | | 54.44 | |
|  | 48h | 1,937,564 | | 1,156,895 | | 59.71 | 1,026,910 | | | 53 | |
|  | Total | 11,217,520 | | 7,016,188 | | 60.81 | 6,207,206 | | | 53.93 | |
| Rep 2 |  |  | |  | |  |  | | |  | |
|  | *S. sclerotiorum* | 708,336 | | 520,981 | | 73.55 | 443,386 | | | 62.6 | |
|  | 1h | 834,200 | | 441,343 | | 52.91 | 396,692 | | | 47.55 | |
|  | 3h | 868,250 | | 526,234 | | 60.61 | 474,374 | | | 54.64 | |
|  | 6h | 2,004,794 | | 1,216,407 | | 60.67 | 1,073,478 | | | 53.55 | |
|  | 12h | 1,900,228 | | 1,123,755 | | 59.14 | 994,114 | | | 52.32 | |
|  | 24h | 1,250,450 | | 663,567 | | 53.07 | 569,734 | | | 45.56 | |
|  | 48h | 1,750,276 | | 1,134,031 | | 64.79 | 996,268 | | | 56.92 | |
|  | Total | 9,316,534 | | 5,626,318 | | 60.68 | 4,948,046 | | | 53.31 | |
| Rep 3 |  |  | |  | |  |  | | |  | |
|  | *S. sclerotiorum* | 3,059,020 | | 2,189,731 | | 71.58 | 1,936,758 | | | 63.31 | |
|  | 1h | 3,667,108 | | 2280561 | | 62.19 | 2,034,016 | | | 55.47 | |
|  | 3h | 2,294,968 | | 1,451,796 | | 63.26 | 1,289,402 | | | 56.18 | |
|  | 6h | 1,673,268 | | 1,010,817 | | 60.41 | 883,100 | | | 52.78 | |
|  | 12h | 1,918,732 | | 1,186,506 | | 61.84 | 1,046,994 | | | 54.57 | |
|  | 24h | 3,835,786 | | 2,267,534 | | 59.12 | 1,984,244 | | | 51.73 | |
|  | 48h | 3,227,198 | | 1,867,826 | | 57.88 | 1,659,720 | | | 51.43 | |
|  | Total | 19,676,080 | | 12,254,771 | | 62.33 | 10,834,234 | | | 55.07 | |
