# Supplementary figures and images for: Changes in the Sclerotinia sclerotiorum transcriptome during infection of Brassica napus
Source: BMC Genomics. 2017 Mar 29;18:266. doi: 10.1186/s12864-017-3642-5 (PMC5372324; doi:10.1186/s12864-017-3642-5)

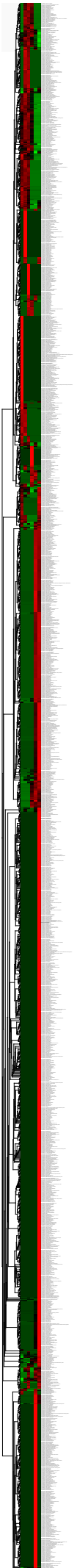

Supplement: Supplementary file 4 — Clustering of differentially expressed genes based on expression patterns relative to time of inoculation (time 0). Increasing intensity indicates greater fold change (red = positive; green = negative) for individual genes. The expression of Sclerotinia sclerotiorum genes at various times during infection of Brassica napus were used for cluster analysis and a heat map was generated. (PDF 208 kb) [file 12864_2017_3642_MOESM4_ESM.pdf]
